# A Reductive Aminase Switches to Imine Reductase Mode for a Bulky Amine Substrate

**DOI:** 10.1021/acscatal.2c06066

**Published:** 2023-01-12

**Authors:** Amelia K. Gilio, Thomas W. Thorpe, Alex Heyam, Mark R. Petchey, Balázs Pogrányi, Scott P. France, Roger M. Howard, Michael J. Karmilowicz, Russell Lewis, Nicholas Turner, Gideon Grogan

**Affiliations:** † Department of Chemistry, University of York, Heslington, York YO10 5DD, U.K.; ‡ School of Chemistry, Manchester Institute of Biotechnology, University of Manchester, 131 Princess Street, Manchester M1 7DN, U.K.; § Pfizer Worldwide Research and Development, 445 Eastern Point Road, Groton, Connecticut 06340, United States

**Keywords:** biocatalysis, imine reductase, reductive aminase, chiral amine, NADPH

## Abstract

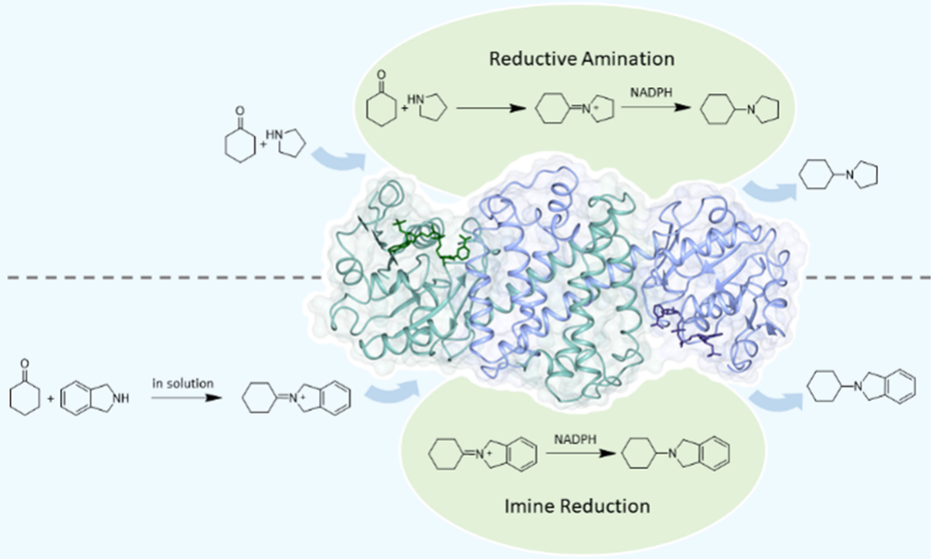

Imine reductases (IREDs) catalyze the asymmetric reduction of cyclic imines, but also in some cases the coupling of ketones and amines to form secondary amine products in an enzyme-catalyzed reductive amination (RedAm) reaction. Enzymatic RedAm reactions have typically used small hydrophobic amines, but many interesting pharmaceutical targets require that larger amines be used in these coupling reactions. Following the identification of IR77 from *Ensifer adhaerens* as a promising biocatalyst for the reductive amination of cyclohexanone with pyrrolidine, we have characterized the ability of this enzyme to catalyze couplings with larger bicyclic amines such as isoindoline and octahydrocyclopenta(*c*)pyrrole. By comparing the activity of IR77 with reductions using sodium cyanoborohydride in water, it was shown that, while the coupling of cyclohexanone and pyrrolidine involved at least some element of reductive amination, the amination with the larger amines likely occurred ex situ, with the imine recruited from solution for enzyme reduction. The structure of IR77 was determined, and using this as a basis, structure-guided mutagenesis, coupled with point mutations selecting improving amino acid sites suggested by other groups, permitted the identification of a mutant A208N with improved activity for amine product formation. Improvements in conversion were attributed to greater enzyme stability as revealed by X-ray crystallography and nano differential scanning fluorimetry. The mutant IR77-A208N was applied to the preparative scale amination of cyclohexanone at 50 mM concentration, with 1.2 equiv of three larger amines, in isolated yields of up to 93%.

## Introduction

**Scheme 1:**
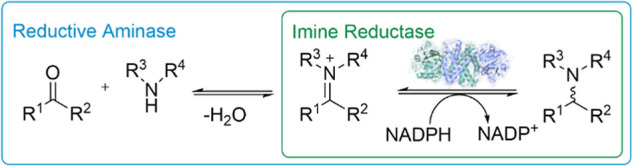
Imine Reduction and Reductive Amination Reactions Catalyzed by IREDs and RedAms

In recent years, the emergence of imine reductases (IREDs) has had a substantial impact on the application of enzymes in the preparation of chiral amines of pharmaceutical interest.^1−5^ IREDs are NAD(P)H-dependent oxidoreductases that catalyze the asymmetric reduction of prochiral imines, but in certain cases also enable the reductive amination of prochiral ketones through catalysis of both imine formation and subsequent reduction of the iminium ion intermediate. In such cases where evidence has been provided of the enzyme catalyzing both chemical steps at near or equimolar ratios of ketone and amine substrates, this subset of IREDs have also been termed “reductive aminases” (RedAms) (Scheme 1).^6−12^

**Scheme 2:**
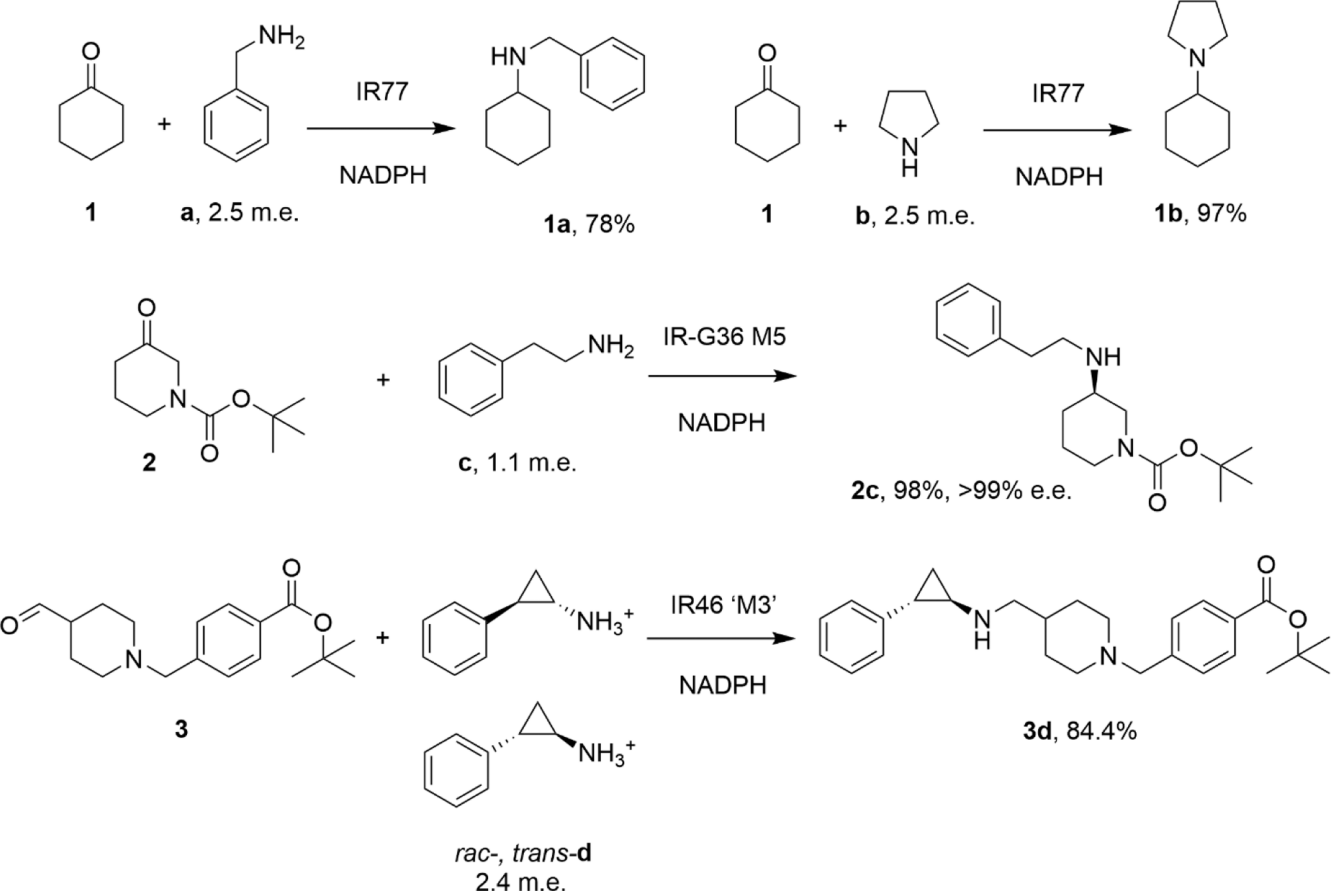
IREDs in Reductive Amination Reactions Using Bulky Amines.^12,17,18^

The fundamental nature of the reductive amination reaction to the formation of amines has ensured that IRED-based technology has now been applied in the pharmaceutical sector at up to kg^13^ and ton^14^ scale. Their ease of expression and application, in a mode similar to well-studied keto-reductases,^15^ has also ensured that IREDs and RedAms have been successfully applied in cascades of up to three enzymes in the synthesis of chiral amines.^10,16^ In the first descriptions of IREDs catalyzing reductive aminations at low amine equivalents,^7^ the amine moiety was in most cases small and hydrophobic, including, for example, methylamine, allylamine, and cyclopropylamine. This meant that while “reductive amination” reactions could certainly be achieved using IREDs with large excesses of amine to favor imine formation in solution, true enzyme-catalyzed reductive aminations at ketone: amine equivalents at or close to 1:1 could for the most part only be achieved with this limited range of amine partners. As part of these initial reports, however, and in publications since, large screens of IREDs from a range of organism sources, including metagenomes,^5^ have revealed enzymes competent for catalyzing reductive amination reactions between selected ketones and larger amine partners, including aniline,^17,18^ benzylamine,^17,18^ and longer aryl alkylamines.^12^ For example, France and co-workers showed that IRED “IR77” catalyzed the amination of cyclohexanone **1** with benzylamine **a** and pyrrolidine **b**, provided at 2.5 molar equiv (m.e.), to give the amine products **1a** and **1b** in up to 78 and 97% conversion, respectively (Scheme 2).^17^ More recently, Gao and co-workers showed that engineered mutants of IR-G36 enabled the amination of *N*-Boc-3-piperidinone **2** with 1.1 equiv of amines such as 2-phenylethylamine **c** to give products such as **2c** with 98% conversion and >99% e.e.^12^

**Scheme 3:**
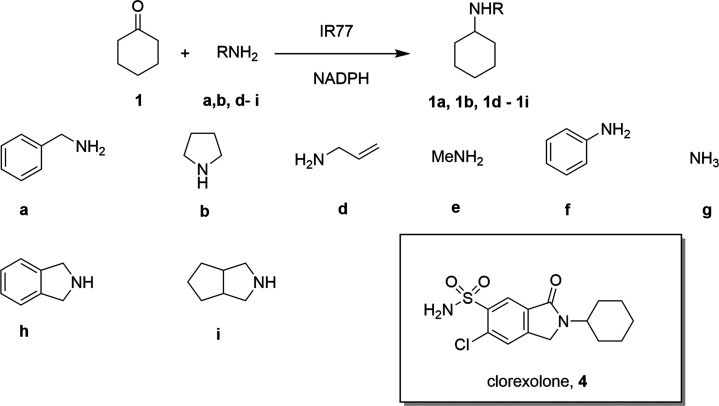
Reductive Amination Substrates Used in This Study

Researchers at GSK also showed that aldehyde **3** could be coupled with amine **d** at 2.4 m.e. using the “M3” variant of “IR46” from *Saccharothrix espanaensis* to give the amine product **3d** in 84.4% yield.^13^ Following the earlier work of our groups,^17^ we were interested to explore the limits of acceptance of bulky amines in the amination of cyclohexanone by “IR77”. In addition to more standard amine partners such as ammonia **g**, methylamine **e** and allylamine **d** (Scheme 3), we were particularly interested in the use of bulky secondary amines such as isoindoline **h**, and octahydrocyclopenta(*c*)pyrrole **i**, as when coupled with cyclohexanone **1**, the products feature scaffolds encountered in pharmaceutical molecules such as the sulfonamide diuretic clorexolone **4** (Scheme 3).

To this end, in this study, we have investigated the ability of IR77 to enable the reductive amination of cyclohexanone with bulkier monocyclic and bicyclic imine substrates. A comparison of IR77-catalyzed transformations with abiotic reductive aminations in which the ketone and amine partners were incubated with sodium cyanoborohydride suggests that, while imine formation between either pyrrolidine or benzylamine with cyclohexanone is likely catalyzed by IR77, reactions with the larger amines are probably a result of the recruitment of imine from solution, followed by reduction. The structure of IR77 was determined, and rational mutagenesis was used to create mutants of improved activity. These have been characterized using kinetics, X-ray crystallography, and nano-differential scanning fluorimetry (nano-DSF), and alanine 208 was identified as a hotspot for improved activity, with mutants A208S and A208N displaying greater catalytic efficiency than the wild-type enzyme.

## Results and Discussion

### Reductive Aminations Using Purified IR77

**Figure 1:**
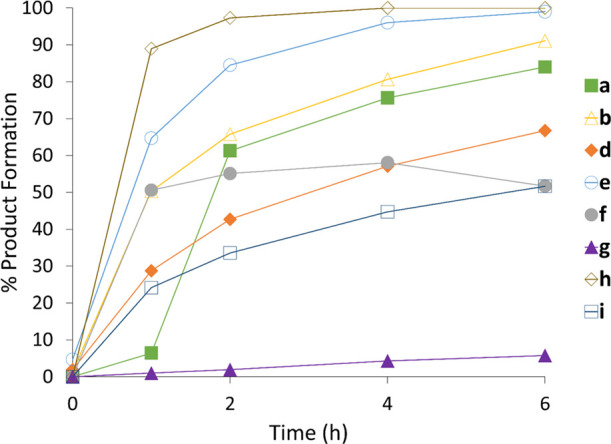
Biotransformations of cyclohexanone **1** with amines **a**, **b,** and **d–i** using purified wt-IR77.

The enzyme designated IR77 by France and co-workers in a previous work^17^ has 100% sequence identity (Figure S1) with NCBI accession sequence WP_053252429, annotated as an “NADP-binding domain” from the nitrogen-fixing bacterium *Ensifer adhaerens*, also known as a species of *Sinorhizobium*. IR77 had previously been shown to be a promising target for reductive amination reactions, giving 78 and 97% yields for the coupling of cyclohexanone **1** with benzylamine **a** and pyrrolidine **b,** respectively, when using cell-free extracts containing the expressed enzyme.^17^ As a first step in investigating the engineering of IR77, the gene was subcloned into the pET-YSBLIC-3C vector,^19^ equipping the protein with an N-terminal hexahistidine tag, and was then expressed in *Escherichia coli* and purified using nickel affinity chromatography followed by size exclusion (Figure S2). The purified enzyme was assayed for activity by gas chromatography analysis in small-scale (3 mL) biotransformations containing 10 mM cyclohexanone **1** with 25 mM amines benzylamine **a**, pyrrolidine **b**, allylamine **d**, methylamine **e**, aniline **f**, or 2 M ammonia **g** (Scheme 3 and Figure 1). In each case, the conversions were superior to those observed with the cell extracts performed previously,^17^ presumably attributable to the higher specific activity of the purified enzyme employed in these reactions. After 6 h, near quantitative conversions to secondary amine products were achieved for reactions with benzylamine **a** and pyrrolidine **b** and methylamine **e**, although poor conversion (<10%) was observed with ammonia **g**, in agreement with previous observations.^17^

Kinetic analysis on wt-IR77 was performed using UV spectrophotometry to monitor the oxidation of NADPH at increasing concentrations of cyclohexanone **1**or pyrrolidine **b** in the presence of a constant concentration of the other (Tables 1, S3, S4 and Figure S5A,B).

**Table 1:** Kinetic Measurements for IR77 Variants with Cyclohexanone **1** and Pyrrolidine **b**

	*k*_cat_ (s^–1^)	*K*_m_ (mM)	*k*_cat_/*K*_m_ (s^–1^ mM^–1^) × 10^–2^	*k*_cat_ (s^–1^)	*K*_m_ (mM)	*k*_cat_/*K*_m_ (s^–1^ mM^–1^) × 10^–2^
variant	**1**	**1**	**1**	**b**	**b**	**b**
wt-IR77	0.24	15	1.6	0.12	17	0.72
A119S	0.25	16	1.6	0.09	23	0.40
C192I	0.33	16	2.1	0.10	9.0	1.1
A208S	0.43	9.6	4.5	0.21	9.7	2.2
A208N	0.50	26	2.0	n.d.	n.d.	n.d.

A *k*_cat_ of 0.24 s^–1^ for cyclohexanone **1**, measured in the presence of pyrrolidine **b**, was between those recorded for the fungal reductive aminases, for example, *Asp*RedAm and *At*RedAm, for which values of 1.47 and 0.11 s^–1^ were previously recorded for the same substrate.^7,8^ The *K*_m_ was 15 mM, significantly higher than values recorded for *Asp*RedAm and *At*RedAm of 1.9 and 2.1 mM, respectively.^7,8^ For pyrrolidine **b**, the *k*_cat_ and *K*_m_ recorded in the presence of 10 mM cyclohexanone were 0.12 s^–1^ and 17 mM. These values were higher and lower, respectively, than those obtained for this amine with *Asp*RedAm (0.08 s^–1^ and 25.1 mM), giving an approximately two-fold improved catalytic efficiency for IR77 over that enzyme for this transformation.^7^

IR77 was also assessed for its ability to catalyze the reductive amination of 10 mM cyclohexanone **1** with 2.5 m.e. of target amines isoindoline **h**, and octahydrocyclopenta(*c*)pyrrole **i**. In these reactions (Figure 1), once again, we observed quantitative conversion to secondary amine products within 2 and 8 h, respectively. Interestingly, kinetics experiments using increasing concentrations of isoindoline **h** and 10 mM cyclohexanone revealed a higher *k*_cat_ of 0.30 s^–1^ and a lower *K*_m_ of 0.61 mM for the bulkier amine (Figure S5C and Table S5), giving a catalytic efficiency of 0.49 s^–1^ mM^–1^, 66-fold higher than for pyrrolidine.

We hypothesized that, rather than this being due to exceptional improvements in binding of **h** to the enzyme for reductive amination, it was reflective rather of a greater amount of intermediate imine that is formed in solution and recruited for reduction by the enzyme directly. This would correspond to a switch in catalytic mode from a reductive aminase in which both imine formation and imine reduction are catalyzed by the enzyme, to an IRED, where the imine is formed in solution and recruited for reduction by the enzyme’s active site.

**Figure 2:**
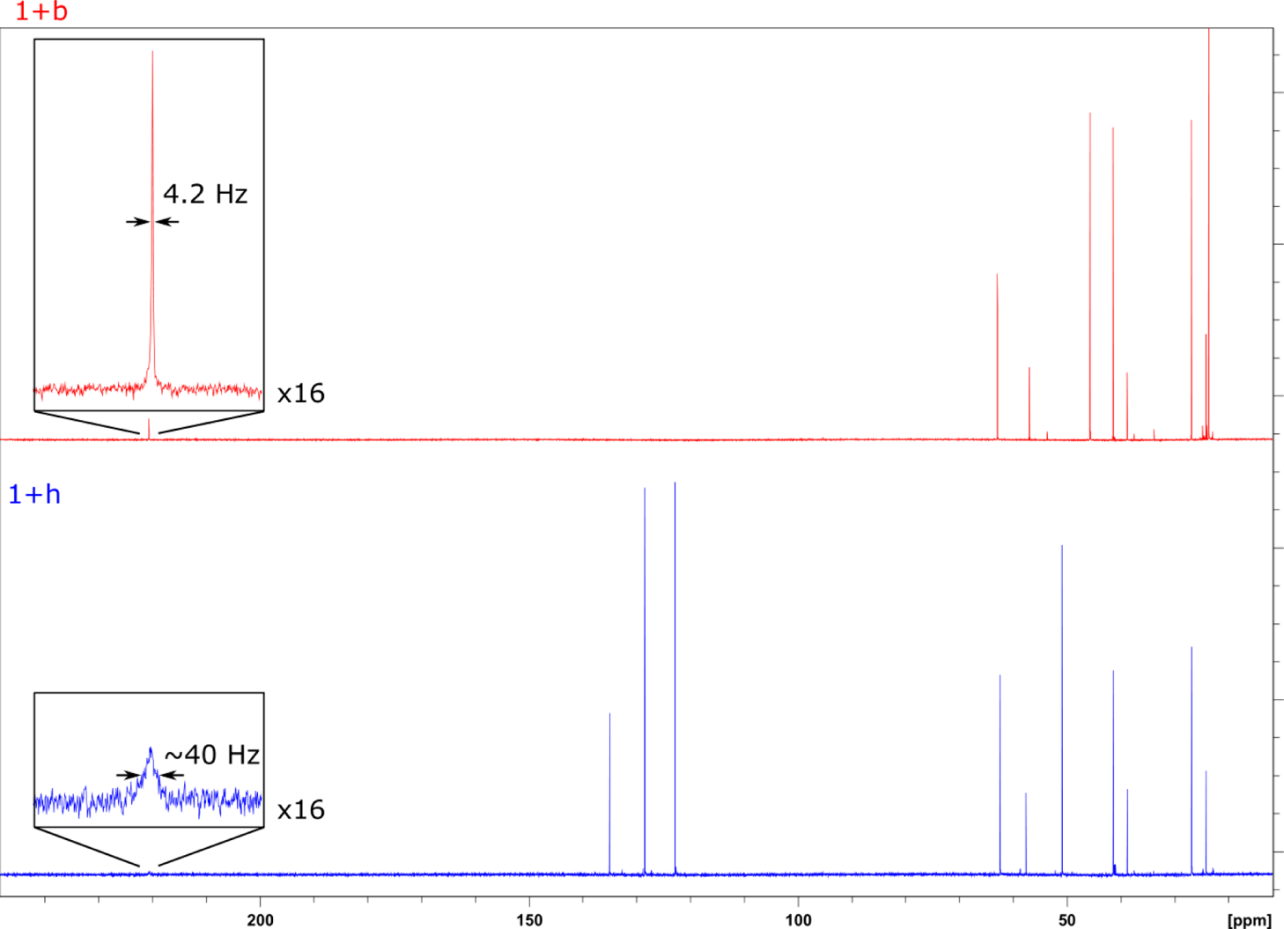
Carbon NMR spectra with water suppression showing cyclohexanone **1** and either pyrrolidine **b** or isoindoline **h** in Tris–HCl buffer at pH 9.0. Enlarged spectra show the carbonyl peak and have 16x vertical magnification. The full width at half maximum of the carbonyl peak is shown. Both spectra were processed with 1 Hz line broadening.

To examine this further, evidence for levels of imine formation in solution was sought by studying mixtures containing **1** and either **b** or **h** in the absence of enzyme by ^13^C NMR (Figure 2).

In the mixture of cyclohexanone **1** with isoindoline **h**, significant broadening of the ketone carbonyl peak at approximately 220.5 ppm was observed with a width of approximately 40 Hz, much wider than all other peaks in spectrum with approximate values of 3 Hz (Figure 2). This suggests that an intermediate is formed in solution during this reaction. However, the lack of peak broadening in the reaction of **1** with **b**, with a much narrower peak width of approximately 4 Hz, suggests that no such intermediate is formed in that case. Although the intermediate suggested by analysis of the reaction between **1** and **h** cannot be characterized by variable temperature experiments due to its short lifetime, its presence suggests that a significant amount of cyclohexanone reacts to form an intermediate in solution in the absence of enzyme.

**Figure 3:**
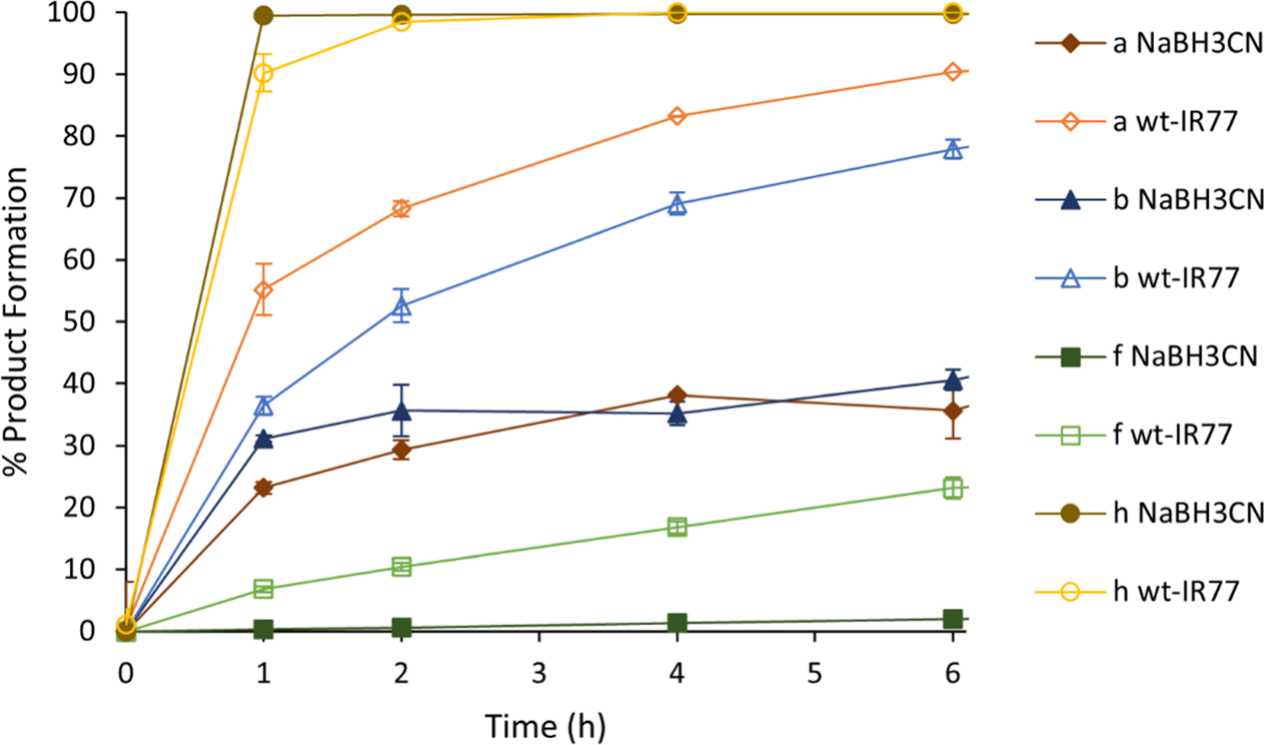
Reaction schemes for cyclohexanone **1** and amines **a**, **b**, **f,** and **h** in the presence of NaBH_3_CN (closed marker) wt-IR77 (open marker).

We had previously compared the reactions catalyzed by RedAms and IREDs with the reductive amination reaction of sodium cyanoborohydride when challenged with a mixture of ketone and amine.^8^ In those experiments, we showed that both *Ad*RedAm and *At*RedAm catalyzed the formation of *N*-allylcyclohexylamine from **1** and **d** much faster than either NaBH_3_CN or an IRED under the same conditions, indicative of a reductive amination mode of action by *Ad*RedAm and *At*RedAm. A similar approach was employed here: we incubated NaBH_3_CN, with **1** and either **a**, **b**, **f,** or **h,** and compared reactions rates with those incubated with wt-IR77 (Figure 3).

The reaction of **1** with isoindoline **h** and NaBH_3_CN displayed similar rates and conversions to that observed with wt-IR77. The results suggest that the relevant imine is formed ex situ and only the imine reduction is catalyzed by IR77. Conversely, reactions with **1** and amines **a**, **b,** and **f** displayed lower rates and conversions with NaBH_3_CN than with wt-IR77, suggesting that both the imine formation and its reduction are catalyzed by IR77 in this case, by a reductive aminase mode. The results suggest that the mode of catalysis of IR77 is therefore dependent on the nature of the amine partner in the reductive amination reaction, including size and its nucleophilicity in water,^20^ which may have a significant influence on the amount of imine formed in solution.

### Structure of IR77

**Figure 4:**
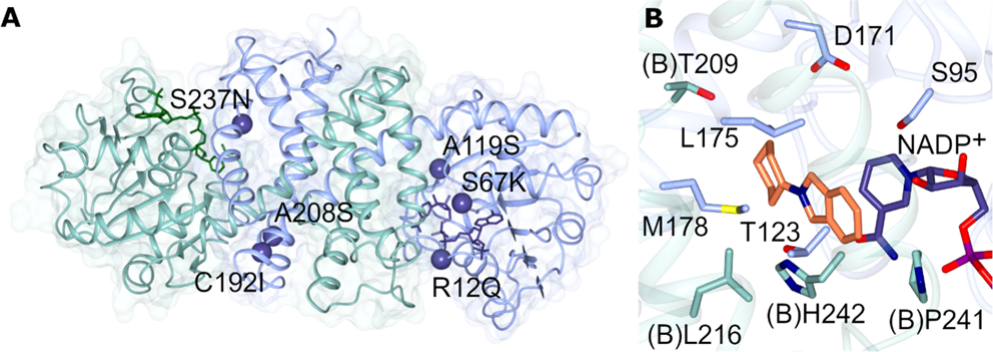
(A) Structure of IR77 dimer with the backbone of subunits A and B shown in blue and green, respectively; the locations of residues selected for mutation are highlighted with blue spheres and annotated; (B) detail of active site of IR77 with backbone and side chain carbon atoms of subunits A and B shown in blue and green, respectively. 2-Cyclohexylisoindoline **1h** was modeled into the active site using Autodock VINA.^27^

As a first step to making mutants of IR77 with improved activity toward **1** with **b** and **h**, the structure of the enzyme was determined by X-ray crystallography and refined to a resolution of 2.58 Å (PDB code 8A3X). Data collection and refinement statistics can be found in Table S6. Crystals were obtained in the *P*2_1_ space group and featured four molecules, representing two dimers, in the asymmetric unit. The enzyme adopts the canonical fold now well established for IRED structures.^7,21−23^ An N-terminal Rossmann domain (residues 1–164) connects to a C-terminal helical domain (195–294) through a long alpha-helix (165–194). Two monomers closely associate in a domain swapping arrangement that serves to form two active sites per dimer, each at the interface of one N-terminal domain with the C-terminal domain of its partner (Figure 4A).

A comparison of the monomer with structures in the PDB using the DALI server^24^ revealed closest structural similarity with the IREDs from *Myxococcus stipitatus* (6TOE; 51% sequence identity; rmsd 1.3 Å over 291 Cα atoms)^25^ and *Streptosporangum roseum* (5OCM; 34%; 1.8 Å over 290).^26^ Following building of the protein atoms and water molecules, clear electron density was observed within each active site that could in each case be modeled as NADP^+^. When multiple dimers have been obtained within the asymmetric unit of IREDs, it has proved possible to discern different conformations within the same structure, representative of “open” and “closed” dimers that hint at the structural flexibility of the IRED during catalysis.^8^ In IR77, each dimer can be considered to be a “closed” structure, with a hydrophobic pocket formed at the cofactor binding site. The substrate binding site is defined by the frequently observed aspartate residue D171, which protrudes from the roof of the cavity into the active site from the interdomain helix (Figure 4B). Facing the nicotinamide ring of the cofactor, on the opposite side of the active site, are the side chains of M178, W179, and H242 from the partner monomer. At the back of the active site are V212 and L216 from the partner monomer and T123. Two features of the active site which could explain the activity of the wild-type IR77 with bulkier imines are the presence of T209 and P241 at the back and front of the binding site as viewed in Figure 4B, which replace much larger tryptophan and methionine residues in IREDs that lack the ability to react with bulkier substrates, such as *Asp*RedAm,^7^ and wild-type IR-G36.^12^ (Figure S7). The product 2-cyclohexylisoindoline **1h** was modeled into the active site of IR77 using Autodock Vina (Figure 4B). Superimposition of the model with structures of *Asp*RedAm clearly showed clashes with W210 and M239 in that enzyme with the cyclohexyl and aromatic rings of the substrate.

### Mutation of IR77

In an effort to identify mutational sites that might improve the activity of IR77, the choice of first-round mutations was informed by previous successful engineering studies on IREDs that had improved activity. In one notable example, researchers at GSK had successfully engineered the IRED “IR46” from *S. espanaensis* for the reductive amination of **3** with **d** (Scheme 2) using point site-saturation mutagenesis and shown that some mutations, with possible generic implications, which were more remote from the substrate binding site, were useful for improving the activity of the IRED for that process.^13^ These included Y142S (IR77 A119), thought to introduce a hydrogen bond to the pendant D194 (IR77 D171); L201F and V215I (IR77 M178 and C192), each thought to introduce favorable hydrophobic interactions at the dimer interface; L37Q and V92K (IR77 R12Q and S67K), thought to be involved in cofactor binding or orientation and S258N, thought to have a role in interaction with the substrate (IR77 S237). In addition, the previous paper also identified mutation Q231F, which was thought to again introduce favorable hydrophobic interactions at the dimer. Superimposition of a structural model of IR46 suggested that IR77 has alanine A208 in this position. Hence, we also mutated A208 to serine to create hydrogen-bonding interactions in a hydrophilic pocket containing T133, N156, and Q158 in the partner monomer. Based upon this analysis, IR77 point mutants R12Q, S67K, A119S, M178F, C192I, S237N, and A208S, illustrated on the IR77 dimer shown in Figure 4A, were chosen as starting points for a rational mutagenesis investigation targeting an improved enzyme for the amination of **1** with **b**, **h,** and **i**.

**Figure 5:**
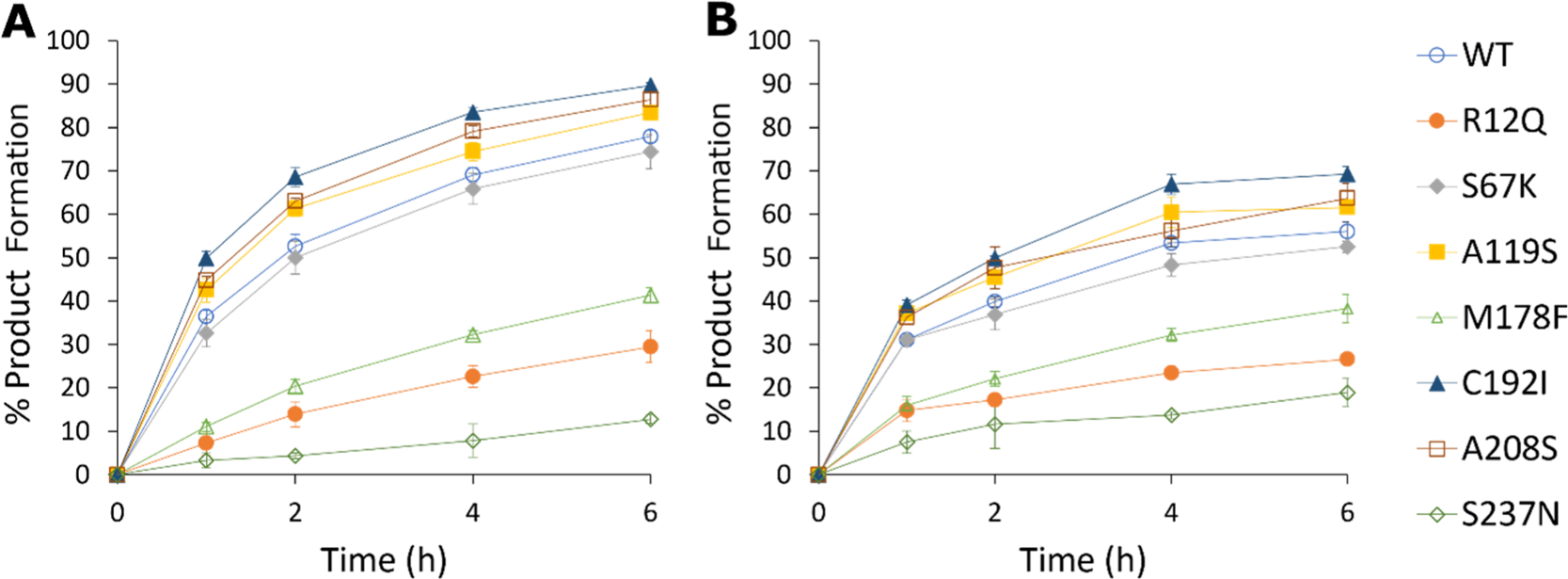
(A) Biotransformations of cyclohexanone **1** with pyrrolidine **b** using mutants of IR77; (B) biotransformations of cyclohexanone **1** with octahydrocyclopenta(*c*)pyrrole **i** using point mutants of IR77.

Point mutants were prepared by InFusion- (Takara Bio)-based mutagenesis starting with PCR using primers listed in Table S2. Mutations were confirmed by sequencing and IR77 muteins prepared and purified as for the wild-type (wt) enzyme. In the first instance, the performance of mutants in the reductive amination of 10 mM cyclohexanone **1** with 25 mM pyrrolidine **b** was assessed using GC analysis (Figure 5A).

These studies revealed that three of the mutants, A119S, C192I, and A208S, clearly catalyzed the reaction at a rate faster than the wt, reaching superior conversions of 74, 83, and 79% compared to the wt (69%) after 4 h. By contrast, active site mutant S237N was the slowest and resulted in the lowest conversion after the same time. M178F, S67K, and R12Q also displayed inferior activity to the wt. In an effort to characterize the basis for the improved activity, A119S, C192I, and A208S were subjected to kinetic analysis using cyclohexanone **1** as the substrate at an invariant concentration of pyrrolidine **b** (Table 1). While the catalytic efficiency (*k*_cat_/*K*_m_) of the A119S and C92I mutants was the same or only marginally higher compared to wt-IR77, that of A208S was 2.85-fold and 2.95-fold increased over the wt for cyclohexanone **1** and pyrrolidine **b,** respectively, attributable both to increases in *k*_cat_ and to a reduction in *K*_m_ in both cases.

Mutants were also assayed with respect to the reductive amination of 10 mM cyclohexanone **1** with 25 mM target amines isoindoline **h**, and octahydrocyclopenta(*c*)pyrrole **i**. For amine **i**, A119S, C192I, and A208S single mutants outperformed wt-IR77 with respect to conversion reaching higher conversions of 62, 69, and 64% within 6 h with octahydrocyclopenta(*c*)pyrrole **i** compared to 56% for wt-IR77 (Figure 5B). Isoindoline **h** proved to be an exceptional amine partner with both the wt-IR77 and best mutants reaching product conversions of >99% within 2 h (Figure S6B). Indeed, at this concentration of **h**, it was not possible to discriminate between the activities of variants for this amine substrate.

The superior performance of the three point mutants identified in the first round prompted us to create a further library of double (A119S/A208S, A119S/C192I, and C192I/A208S) and triple (A119S/C192I/A208S) mutants that combined the beneficial mutations. Interestingly, the combination of mutations was, for most muteins, largely not additive; indeed, the catalytic performance of most in the reductive amination of cyclohexanone **1** with pyrrolidine **b** was inferior to that of wt-IR77 or single-point mutants (Figure S6). The triple mutant A119S/C192I/A208S was the poorest of the combined mutants overall. However, in the case of A119S/A208S, conversion after 1 h was slightly improved from 36% (wt) to 41% (A119S) and 45% (A208S) to 52% (Figure S6D), although kinetic analysis showed that the catalytic efficiency of this double mutant was inferior to that of wt-IR77, with a *k*_cat_/*K*_m_ of 0.23 s^–1^ mM^–1^.

**Figure 6:**
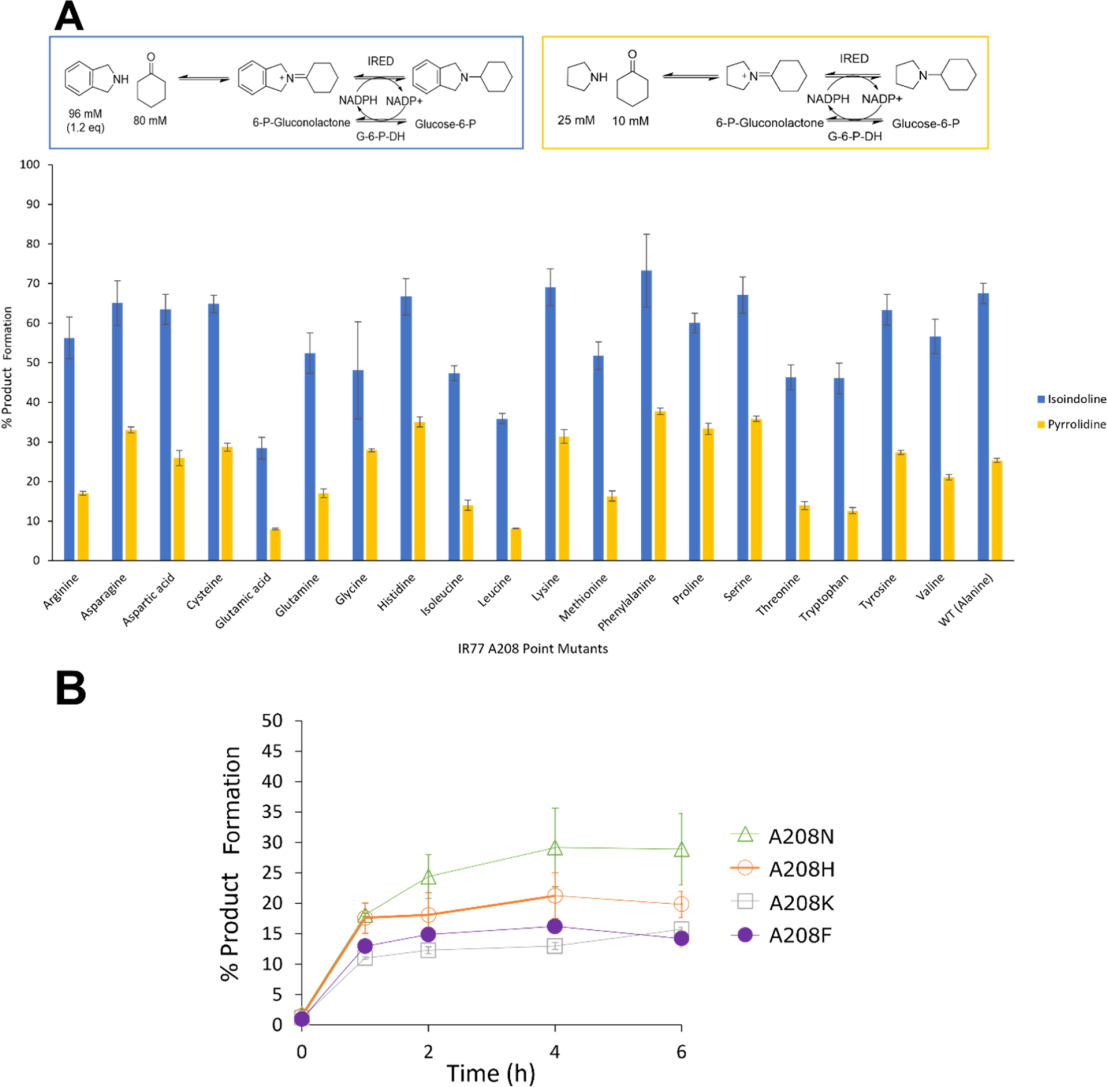
(A) Biotransformations with A208 SSM library of IR77. (B) Biotransformations with best four mutants from SSM library at a higher substrate loading.

Given the lack of improved activity in the combined mutants, the site of mutation with the best catalytic efficiency, A208S, was further examined using a site saturation mutagenesis (SSM) library. Preliminary 30 min reactions with **1** and **b** or **h** with all 19 point mutants filtered out those with the lowest conversions, highlighting seven with conversions greater than the wt-IR77 for further testing: A208N, A208D A208C, A208H, A208F, A208K, and A208Y (Figure 6A). Reactions with 80 mM **1** and 1.2 m.e. **h** permitted the identification of four mutants (A208N, A208H, A208K, and A208F) with superior conversion over 24 h compared to A208S. In order to discriminate the performance of these mutants, substrate loadings were increased to 150 mM cyclohexanone and 1.2 m.e. isoindoline. Although conversions plateaued after around 4 h due to the high substrate loadings, the best performance was observed with A208N, reaching 30% conversion compared to 22, 15, and 16% for A208H, A208K, and A208F, respectively (Figure 6B).

The catalytic efficiency of A208N was evaluated with kinetic measurements (Table 1 and Figure S5). A *k*_cat_ of 0.50 s^–1^ for cyclohexanone **1**, measured in the presence of pyrrolidine **b**, was greater than that recorded for wt-IR77 (0.25 s^–1^) and A208S (0.44 s^–1^) (Figure S5A). However, the *K*_m_ of 26 mM was greater than those recorded for wt-IR77 (16 mM) and A208S (9.6 mM), leading to a catalytic efficiency only 1.24 fold greater than the wild type and approximately half that of A208S. For isoindoline **h**, the *k*_cat_ recorded in the presence of 10 mM cyclohexanone was 0.28 s^–1^, in between those recorded for wt-IR77 and A208S of 0.30 and 0.23 s^–1^, respectively (Figure S5C). However, the *K*_m_ of 0.94 mM was greater than those recorded for wt-IR77 and A208S of 0.61 and 0.60 mM, giving 60% of the catalytic efficiency of wt-IR77 in this case. The lower catalytic efficiency of A208N over A208S is compensated for by the greater stability observed by the former. An increase in melting temperature (*T*_m_), measured using nano-DSF, of 8.2 °C was observed upon binding of cofactor, over 2 °C greater than the difference observed with wt-IR77 and 1 °C greater than A208S (Figure S8). This improvement in stability is illustrated in the capability of IR77 A208N to withstand significantly greater substrate loadings than either wt-IR77 or A208S.

### Structure of IR77 A208N

**Figure 7:**
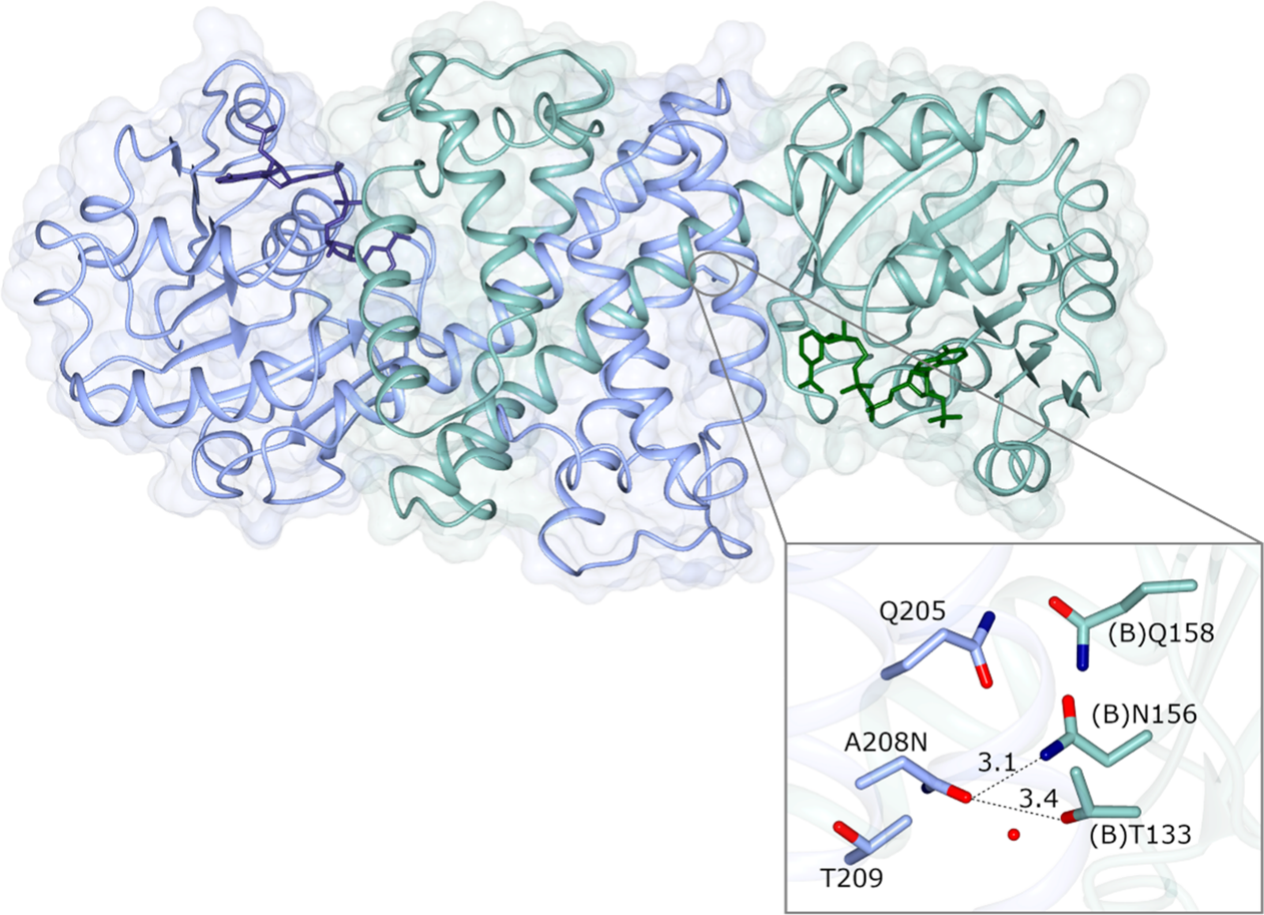
Structure of IR77 A208N mutant (8A5Z) highlighting interdimer interactions established as a result of mutating position A208 to asparagine (inset). Distances are given in Ångstroms.

To shed light on molecular interactions that may affect the activity and stability of the A208N mutant, its structures were also determined using X-ray crystallography (PDB code 8A5Z). Crystals of IR77 A208N were obtained in the *P*2_1_2_1_2_1_ space group with two molecules in the asymmetric unit constituting one dimer. The dimer featured one more open active site and the other more closed, and more resembling the conformation observed with those in the wild-type structure. Following building and refinement of the structure, electron density for the cofactor NADP^+^ was again clearly visible within the active sites at each dimer interface. Position A208N is observed at the interface of the C-terminal helical domain of one monomer and the N-terminal domain of its partner. Introduction of the mutation has resulted in new H-bonding interactions between the N208 side chain and those of T133 and N156 in the partner monomer (Figure 7). Interestingly, Asn is also found in this position in the IRED from *Actinoalloteichus hymeniacidonis* reported by Gao and co-workers (PDB 7WNN),^12^ although in that instance, there are no H-bonding side chains on the partner monomer to form stabilizing interactions. However, the benefit of engineering intersubunit interactions in IREDs for improved stability and activity previously described^12,13^ is consolidated by the structural data on IR77-A208N in conjunction with the biotransformation experiments.

### Preparative Reductive Aminations by IR77-A208N

To assess the applicability of these reactions with the best mutein, IR77 A208N, preparative scale reactions were performed on approximately 100 mg scale in 20 mL buffer with substrate loadings of 50 mM cyclohexanone and 1.2 m.e. of benzylamine **a**, pyrrolidine **b,** and isoindoline **h**. Substrate loadings were selected to maintain a balance between high loadings and high product formation. Products were isolated with good yields of 71, 62, and 93%, respectively, after flash column purification, highlighting the potential synthetic capability of IR77 A208N at higher concentrations of substrate.

## Conclusion

In summary, we report the application of a wild-type IRED for the reductive amination of ketones with bulky amines. IR77 is an enzyme capable of tolerating high substrate loadings of up to 150 mM and low ketone: amine equivalents of 1:1.2 with cyclohexanone and isoindoline. We have provided further evidence that bulky amines can be used as amine donors in reductive amination reactions to give products with high yields, but that some larger amines, with greater nucleophilicity in water, may be processed in a different catalytic mode to that of smaller amines, with ex situ imine formation and subsequent enzyme-catalyzed reduction predominating in these cases. We have also shown that mutations that target improved IRED performance through the formation of new intermolecular interactions can lead to mutants of superior process stability, an approach that may be transferred to other IRED engineering studies in the future.

## Experimental Section

For full details of experimental procedures, see Supporting Information.

## Supporting Information Available

Cloning and expression of IR77 genes and protein purification, mutagenesis of IR77, determination of kinetic constants, biotransformations, preparative-scale biotransformations, GC analysis, time courses of biotransformations analysed using GC, synthesis of standards and characterisation of reaction products, syntheses of amine standards, NMR analysis of imine formation in solution, structural alignment with AspRedAm, autodock models, and nano DSF measurements (PDF)
