# Supplementary material for: A Reductive Aminase Switches to Imine Reductase Mode for a Bulky Amine Substrate
Source: ACS Catal. 2023 Jan 12;13(3):1669–77. doi: 10.1021/acscatal.2c06066 (PMC9903292; doi:10.1021/acscatal.2c06066)
Supplement: Supplementary file 1 — cs2c06066_si_001.pdf [file cs2c06066_si_001.pdf]

A Reductive Aminase Switches to Imine Reductase Mode for a Bulky Amine Substrate

Amelia K. Gilio,[a] Thomas W. Thorpe,[b] Alex Heyam,[a] Mark R. Petchey,[a] Balázs Pogrányi,[a] Scott P. France,[c] Roger M. Howard,[c] Michael J. Karmilowicz,[c] Russell Lewis,[c] Nicholas Turner[b] and Gideon Grogan[a]*

[a]Department of Chemistry, University of York, Heslington, York YO10 5DD UK

[b]School of Chemistry, University of Manchester, Manchester Institute of Biotechnology, 131 Princess Street, Manchester, M1 7DN, U.K.

[c]Pfizer Worldwide Research and Development, 445 Eastern Point Road, Groton, Connecticut 06340, United States

SUPPORTING INFORMATION

*author to whom correspondence should be addressed; Tel: 44 1904 328256; e-mail: gideon.grogan@york.ac.uk

Table of Contents

SUPPORTING INFORMATION 1

1. Cloning and Expression of IR77 Genes and Protein Purification 3
2. Mutagenesis of IR77 6

   Mutagenesis Protocol 6

3. Determination of Kinetic Constants 9
4. Biotransformations 11
5. Preparative-Scale Biotransformations 12
6. GC Analysis 12
7. Time courses of biotransformations analysed using GC 13
8. Synthesis of Standards and Characterisation of Reaction Products 14

General 14

Syntheses of amine standards 1a and 1f 15

Syntheses of amine standards 1b and 1e 16

Syntheses of Amine standards 1h and 1i 18

Characterization of Products from Scaled Biotransformations 19

9. $NaBH_3CN$ Reactions and Comparisons 21

   Reaction Protocol for reductive amination using $NaBH_3CN$ 21

10. NMR analysis of Imine Formation in Solution 21
11. Crystallisation, Data Collection and Structure Solution and Refinement 21

    Protein crystallisation 21

    Data collection, structure solution and refinement 22

12. Structural Alignment with *Asp*RedAm 24
13. Autodock Models 24
14. Nano DSF measurements 24
15. References 26

1. Cloning and Expression of IR77 Genes and Protein Purification

```
IR46            MGSSHHHHHHSSGLVPRGSHMVTEASPAPVSVIGLGLMGAALAGAYLKAGHQTTVWNRSA
AspRedAm        -------------------------MSKHIGIFGLGAMGTALAAKYLEHGYKTSVWNRTT
IR77            -------------------------MKPSISVLGTGRMGSALARALLQAGYRTVVWNRTS
                                             :.::* * **:***   *: *::* ****::

IR46            GKADALVAQGATNAADIAEAVAASDVLVVCVVDYAAFHAILEP--VKDALQGKVIVNLTS
AspRedAm        AKAIPLVEQGAKLASTISEGVNANDLIIICLLNNQVVEDALRD--ALQTLPSKTIVNLTN
IR77            EKAEPLAALGATVAPTVRQAIDASGIVIVNVSDYAATSTLLRASDVTPGLRGKLIVELTS
                 ** .*.  **. *. : :.: *..:::: : :  .    *.   .   * .* **:**.

IR46            GLPDDARGAAEWASGTGAEYLDGYIMSVPPGVGLPQTLLFYGG-DADVFAKHEATLKVLG
AspRedAm        GTPNQARKLADFVTSHGARYIHGGIMAVPTMIGSPHAVLLYSGESLELFQSIESHLSLLG
IR77            GTPEGARETSQWTAAHGARYLDGAILATPDFIGTDAGTILLSG-ALEPFAANEDVFRALG
                  * *: **  :::.:. **.*:.* *::.*  :*     :: .*   : *   *  :
**

IR46            GNSIHLGADAGVAALYDLGLLAILWSSLAGALHAYALVAS--EK-IPAAALAPFAEQWIT
AspRedAm        -MSKYLGTDAGSASLHDLALLSGMYGLFSGFLHAVALIKSGQDTSTTATGLLPLLTPWLS
IR77            GNVQHIGTEPGLANALDSAVLALMWGALFGGLHAIAVCRAEEIDLGELGRQWAATAPVVE
                    ::*::.* *   * .:*: ::. : * *** *:  :            .     :

IR46            HVVLPSVKGAAAAVDSGQYATSVSTTALNAVGLGKMVEASKAAGIRPDLMLPIKAYLEQR
AspRedAm        AMTG-YLSSIAKQIDDGDYATQGSNLGMQLAGVENIIRAGEEQRVSSQMILPIKALIEQA
IR77            GLVADLIKRTSAGRFVSDAET-LSSISPHYGAFQHLKELMEARRIDRTVVDGYDAIFRRA
                 :.   :.  :     .:  *  *. . :  .. :: .  :   :   ::   .* :.:

IR46            VADGHGEEALAGMFEVIRSPER--
AspRedAm        VGEGHGGEDLSALIEYFKVGKNVD
IR77            IASGHLHDDFAALSQFMGKAEQP-
                :..**  : ::.: : :   :.
```

Figure S1. Amino acid sequence of IR77 from *Ensifer adhaerens* aligned with that of 'IR46' from *Saccharothrix espanaensis*[1] and AspRedAm.[2] The aspartate residue D171, characteristic of this group of IREDs, is boxed in red; the residues chosen for mutation in the first round of mutagenesis are boxed in blue alongside the structurally aligned residues in IR46, or in yellow in the case of Q231 (IR46), where residues were matched based on structural models.

Sequence of codon optimised IR77 WT gene

ATGAAGCCGAGCATTAGCGTTCTGGGTACCGGTCGTATGGGTAGCGCGCTGGCG
CGTGCGCTGCTGCAGGCGGGTTACCGTACCGTGGTTTGGAACCGTACCAGCGAG
AAAGCGGAACCGCTGGCGGCGCTGGGTGCGACCGTTGCGCCGACCGTGCGTCAA
GCGATTGACGCGAGCGGCATCGTGATTGTTAACGTGAGCGATTATGCGGCGACC
AGCACCCTGCTGCGTGCGAGCGATGTTACCCCGGGTCTGCGTGGCAAGCTGATCG
TGGAACTGACCAGCGGTACCCCGGAAGGTGCGCGTGAAACCAGCCAGTGGACCG
CGGCGCACGGTGCGCGTTATCTGGATGGTGCGATCCTGGCGACCCCGGACTTCAT
TGGTACCGATGCGGGTACCATCCTGCTGAGCGGTGCGCTGGAGCCGTTTGCGGCG
AACGAAGACGTTTTTCGTGCGCTGGGTGGCAACGTGCAACACATTGGTACCGAA
CCGGGTCTGGCGAACGCGCTGGATAGCGCGGTTCTGGCGCTGATGTGGGGCGCG
CTGTTTGGTGGCCTGCATGCGATTGCGGTGTGCCGTGCGGAGGAAATCGACCTGG
GTGAACTGGGTCGTCAGTGGGCGGCGACCGCGCCGGTGGTTGAAGGTCTGGTTG
CGGACCTGATTAAGCGTACCAGCGCGGGCCGTTTCGTGAGCGATGCGGAGACCC
TGAGCAGCATCAGCCCGCACTACGGTGCGTTTCAACACCTGAAAGAGCTGATGG
AAGCGCGTCGTATCGATCGTACCGTGGTTGACGGTTATGATGCGATCTTCCGTCG
TGCGATTGCGAGCGGTCACCTGCACGATGATTTTGCGGCGCTGAGCCAGTTTATG
GGTAAAGCGGAACAACCGTAA

The LIC3C plasmid containing the gene for IR77 or a mutant of IR77 was used to transform *E. coli* BL21 (DE3) competent cells for gene expression. Pre-cultures were grown in LB-medium (10 mL) containing 30 μg mL^{-1} kanamycin for 18 h at 37°C with shaking at 180 r.p.m. 1 L volume cultures were inoculated with the pre-culture (10 mL) and incubated at 37°C, with shaking at 180 r.p.m. until an OD600 of 0.6-0.8 was reached. Gene expression was induced by addition of IPTG (1 mM) and shaking was continued overnight at 16°C with shaking at 180 r.p.m. The cells were then harvested by centrifugation at 5000 *g* for 20 min and resuspended in 50 mM Tris-HCl buffer pH 7.1, containing 300 mM NaCl and 10% (v/v) glycerol and cOmplete mini protease inhibitor. Cells were disrupted, and the suspension was centrifuged at 15,000 *g* for 40 min to yield a clear lysate. The N-terminal His6-tagged protein was purified using immobilised-metal affinity chromatography (IMAC) using Ni-NTA column, followed by size exclusion chromatography (SEC) The lysate was loaded onto a pre-equilibrated Ni-

NTA column, followed by washing with a load buffer (50 mM Tris-HCl, 300 mM NaCl, 10% v/v glycerol pH 7.1). The bound protein was eluted using a linear gradient with buffer containing 500 mM imidazole. Fractions were analysed by 12 % acrylamide SDS PAGE (**Figure S2A**). Fractions containing IR77 were pooled, concentrated, and loaded onto a HiLoad 16/600 Superdex 75 gel filtration column pre-equilibrated with buffer containing 50 mM Tris-HCl, 300 mM NaCl and 10% (v/v) glycerol at pH 7.1. Fractions were analysed by 12 % acrylamide SDS PAGE (**Figure S2B**). The concentrated protein sample after gel filtration was used for crystallization screening.

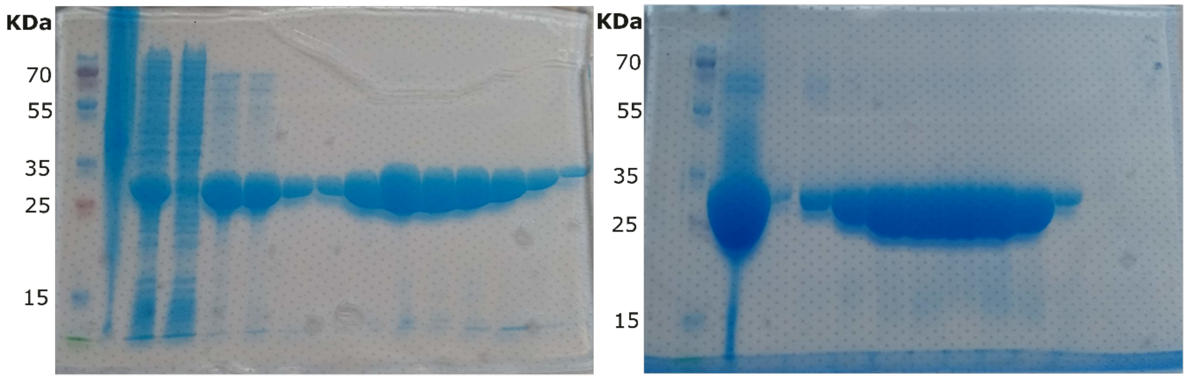

Figure S2. 12% acrylamide SDS PAGE gels of the Ni-NTA column **A** and gel filtration column **B** showing IR77 at approximately 31 kDa.

2. Mutagenesis of IR77

Mutagenesis Protocol

IR77 mutants were generated by using an In-Fusion® HD Cloning Kit (Clontech) or a QuickChange Lightning kit (Agilent) according to the manufacturer's protocol, using complementary and overlapping primers with approximately 15 bp overhangs. Mutant primers are listed in **Table S1** and **Table S2**. Inverse PCRs were performed with KOD Hotstart polymerase (Merck) according to the manufacturer's protocol. PCRs were carried out using a LifeEco thermocycler (Bioer) with annealing temperatures calculated with the Thermofisher Tm calculator (https://www.thermofisher.com/uk/en/home/brands/thermo-scientific/molecular-biology/molecular-biology-learning-center/molecular-biology-resource-library/thermo-scientific-web-tools/tm-calculator.html).

Table S1. PCR primers used for site directed mutagenesis experiments.

**Mutation**	**Primer Sequence 5' to 3'**
R12Q	Fwd: TACCGGTCAAATGGGTAGCGCGCTGGCG<br>Rev: CCCATTTGACCGGTACCCAGAACGCTAATGC
S67K	Fwd: TAACGTGAAGGATTATGCGGCGACCAGC<br>Rev: TAATCCTTCACGTTAACAATCACGATGCCGC
A119S	Fwd: GGATGGTAGCATCCTGGCGACCCCGGAC<br>Rev: AGGATGCTACCATCCAGATAACGCGCACC
M178F	Fwd: GGCGCTGGAATGGGGCGCGCTGTTTGGT<br>Rev: CCCCATTCCAGCGCCAGAACCGCGCTA
C192I	Fwd: TGCGGTGATTCGTGCGGAGGAAATCGACC<br>Rev: GCACGAATCACCGCAATCGCATGCAG
A208S	Fwd: GTGGGCGAGCACCGCGCCGGTGGTTGAAG<br>Rev: GCGGTGCTCGCCCACTGACGACCCAG
S237N	Fwd: GACCCTGAACAGCATCAGCCCGCACTAC<br>Rev: ATGCTGTTCAGGGTCTCCGCATCGCT

Table S2. PCR primers used for site saturation mutagenesis experiments.

A208 Mutant	Primer Sequence 5' to 3'
Arginine (R)	Fwd: GTGGGCGCGCACCGCGCCGGTGGTTGAAG<br>Rev: GCGGTGCGCGCCCACTGACGACCCAG
Asparagine (N)	Fwd: GTGGGCGAACACCGCGCCGGTGGTTGAAG<br>Rev: GCGGTGTTCGCCCACTGACGACCCAG
Aspartate (D)	Fwd: GTGGGCGGATACCGCGCCGGTGGTTGAAG<br>Rev: GCGGTATCCGCCCACTGACGACCCAG
Cysteine (C)	Fwd: GTGGGCGTGCACCGCGCCGGTGGTTGAAG<br>Rev: GCGGTGCACGCCCACTGACGACCCAG
Glutamate (E)	Fwd: GTGGGCGGAAACCGCGCCGGTGGTTGAAG<br>Rev: GCGGTTTCCGCCCACTGACGACCCAG
Glutamine (Q)	Fwd: GTGGGCGCAGACCGCGCCGGTGGTTGAAG<br>Rev: GCGGTCTGCGCCCACTGACGACCCAG
Glycine (G)	Fwd: GTGGGCGGGCACCGCGCCGGTGGTTGAAG<br>Rev: GCGGTGCCCGCCCACTGACGACCCAG
Histidine (H)	Fwd: GTGGGCGCACACCGCGCCGGTGGTTGAAG<br>Rev: GCGGTGTGCGCCCACTGACGACCCAG
Isoleucine (I)	Fwd: GTGGGCGATTACCGCGCCGGTGGTTGAAG<br>Rev: GCGGTAATCGCCCACTGACGACCCAG
Leucine (L)	Fwd: GTGGGCGCTGACCGCGCCGGTGGTTGAAG<br>Rev: GCGGTCAGCGCCCACTGACGACCCAG
Lysine (K)	Fwd: GTGGGCGAAAACCGCGCCGGTGGTTGAAG<br>Rev: GCGGTTTTCGCCCACTGACGACCCAG
Methionine (M)	Fwd: GTGGGCGATGACCGCGCCGGTGGTTGAAG<br>Rev: GCGGTCATCGCCCACTGACGACCCAG
Phenylalanine (F)	Fwd: GTGGGCGTTTACCGCGCCGGTGGTTGAAG<br>Rev: GCGGTAAACGCCCACTGACGACCCAG
Proline (P)	Fwd: GTGGGCGCCGACCGCGCCGGTGGTTGAAG<br>Rev: GCGGTCGGCGCCCACTGACGACCCAG
Threonine (T)	Fwd: GTGGGCGACCACCGCGCCGGTGGTTGAAG<br>Rev: GCGGTGGTCGCCCACTGACGACCCAG
Tryptophan (W)	Fwd: GTGGGCGTGGACCGCGCCGGTGGTTGAAG<br>Rev: GCGGTCCACGCCCACTGACGACCCAG
Tyrosine (Y)	Fwd: GTGGGCGTATACCGCGCCGGTGGTTGAAG<br>Rev: GCGGTATACGCCCACTGACGACCCAG
Valine (V)	Fwd: GTGGGCGGTGACCGCGCCGGTGGTTGAAG<br>Rev: GCGGTCACCGCCCACTGACGACCCAG

Mutant genes were expressed and purified using the same protocol as for the wild-type enzyme described above. SDS-PAGE analysis was performed on lysates obtained after cell disruption to confirm the soluble expression of the mutants. SDS-PAGE gels for the expression of first and second round mutations of IR77, and the saturation mutagenesis library at position 208, are shown in **Figure S3** and **Figure S4** respectively.

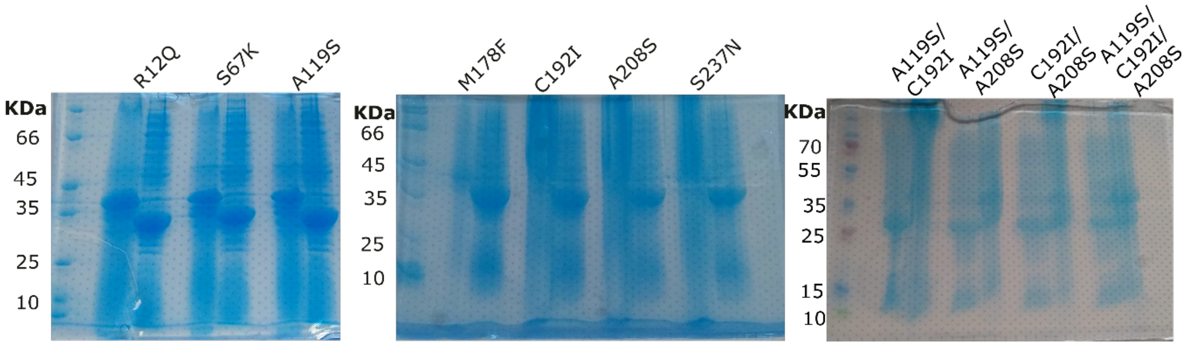

Figure S3. SDS PAGE analysis of IR77 site directed mutant lysates. Insoluble (left) and soluble (right) fractions for each mutant are shown.

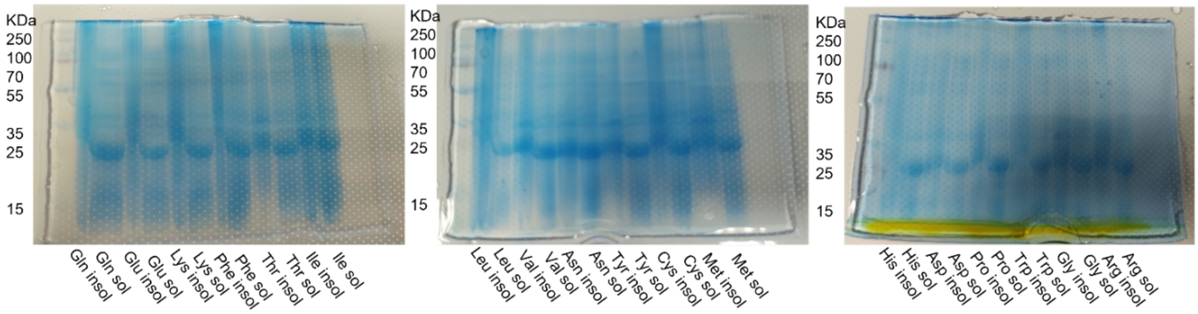

Figure S4. SDS PAGE analysis of IR77 site saturation mutant lysates. Insoluble (left) and soluble (right) fractions for each mutant are shown.

3. Determination of Kinetic Constants

For the determination of kinetic constants for cyclohexanone **1** (**Table S3**, **Figure S5A**), a typical reaction mixture contained 0-80 mM of cyclohexanone **1**, 96 mM pyrrolidine **b** from buffer stock adjusted to pH 9, 0.5 mM NADPH, 1 % (v/v) dimethylsulfoxide and 0.2 mg mL^{-1} of purified IR77 in 1 mL total volume (100 mM Tris-HCl, pH 9). For the determination of kinetic constants for amines pyrrolidine **b** (**Table S4, Figure S5B**), and isoindoline **h** (**Table S5, Figure S5C**), a typical reaction mixture contained 0-120 mM amine, 10 mM cyclohexanone, 0.5 mM NADPH, 1 % (v/v) dimethylsulfoxide and 0.2 mg mL^{-1} of purified IRED in a total volume of 1 mL (100 mM Tris-HCl, pH 9). For the determination of kinetic constants for NADPH a typical reaction mixture contained 0-2 mM NADPH, 10 mM cyclohexanone, 12 mM pyrrolidine, 1 % (v/v) dimethylsulfoxide and 0.2 mg/mL of purified IRED in a total volume of 1 mL (100 mM Tris-HCl, pH 9). Activity measurements were performed in duplicate at 340 nm (ε = 6.22 mM^{-1} cm^{-1}) using a Cary UV-Vis spectrophotometer (Agilent). Kinetic constants were determined through non-linear regression based on Michaelis-Menten kinetics using OriginLab software.

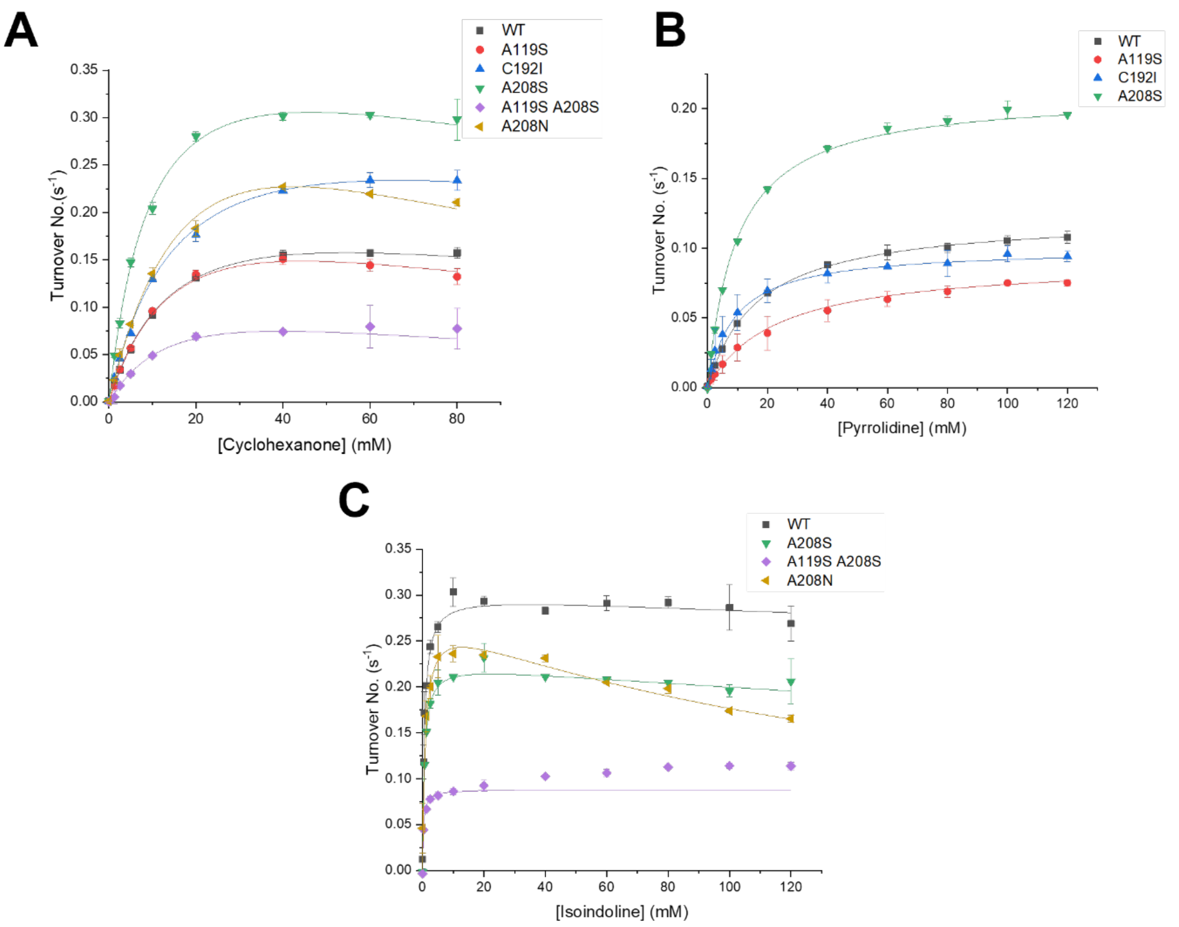

Figure S5. Kinetic plots for IR77 with **A:** cyclohexanone **1, B:** pyrrolidine **b** and **C:** Isoindoline **h.**

Table S3. Kinetic data for wt-IR77 and mutants with cyclohexanone.

	k_{cat} (s^{-1})	K_m (mM)	k_{cat}/K_m (s^{-1} mM^{-1})	Factor Improvement in k_{cat}/K_m
**WT**	0.24	15	0.016	-
**A119S**	0.25	16	0.016	1.0
**C192I**	0.33	16	0.021	1.3
**A208S**	0.43	9.6	0.045	2.9
**A119S/A208S**	0.14	18	0.0078	0.51
**A208N**	0.50	26	0.020	1.2

Table S4. Kinetics data for wt-IR77 and mutants with pyrrolidine.

	k_{cat} (s^{-1})	K_m (mM)	k_{cat}/ K_m (s^{-1} mM^{-1})	Factor Improvement in k_{cat}/ K_m
**WT**	0.12	17.0	0.0071	-
**A119S**	0.09	23.0	0.0039	0.54
**C192I**	0.10	9.00	0.011	1.5
**A208S**	0.21	9.70	0.022	3.0

Table S5. Kinetics data for wt-IR77 and mutants with isoindoline.

	k_{cat} (s^{-1})	K_m (mM)	k_{cat}/K_m (s^{-1} mM^{-1})	Factor Improvement in k_{cat}/K_m
**WT**	0.30	0.61	0.49	-
**A208S**	0.23	0.60	0.37	0.77
**A119S/A208S**	0.089	0.31	0.28	0.57
**A208N**	0.28	0.94	0.30	0.61

4. Biotransformations

In a 10 mL glass vial, cyclohexanone (10 mM), amine (2.5 eq.) and IRED lysate (600 µL from a 100 mg / mL stock solution based on wet cell weight) were added with cofactor, $NADP^+$ (0.40 mM) and a cofactor recycling system of glucose-6-phosphate (G6P, 30 mM) and G6P-dehydrogenase (G6PDH, 2 Units). The reaction was made up to 3 mL with Tris-HCl buffer (100 mM, pH 9.0) and the reaction media was shaken at 250 r.p.m. at 25 ˚C for 24 h. 200 µL aliquots of the reaction mixture were basified with 16 µL NaOH (10 M) after which the aqueous layer was extracted with 400 µL EtOAc and the separated organic phase was dried over $MgSO_4$. The supernatant was directly subjected to gas chromatography (GC) analysis, detection was performed using a flame-ionisation detector (FID). Control reactions (500 µL total volume) without IRED, without cofactor, or without Glucose-6P-dehydrogenase were run alongside and 200 µL aliquots of these were taken at 0 h and 24 h time points.

For reactions with isoindoline, the reaction mixtures were made up in a 10 mL vial and 200 μL fractions were measured into 500 μL Eppendorf tubes immediately after amine addition at 0 h. At each time point an Eppendorf tube was basified and extracted as described in the protocol above.

Reactions with 1.2 eq. and higher amine loadings were performed using the protocol described above.

5. Preparative-Scale Biotransformations

Dimethylsulfoxide stock solution of cyclohexanone (50 mM, 1.0 eq.), amine (1.2 eq.) and IRED cell free lysate (4 mL from a 100 mg / mL stock solution based on wet cell weight) were mixed with cofactor, $NADP^+$ (0.4 mM, 0.0080 eq.) and a cofactor recycling system of G6P (30 mM) and G6PDH (13 Units) in a 50 mL Falcon(R) polypropylene centrifuge tube. The reaction was diluted to 20 mL volume with Tris-HCl buffer (100 mM, pH 9.0). The tubes were shaken at 250 r.p.m. at 25 ˚C for 24 h. After 24h, the reaction was basified with 1.6 mL NaOH (10.0 M), the aqueous layer was extracted with EtOAc (2 x 20 mL), the unified organic phase was dried over $MgSO_4$ and concentrated under reduced pressure. Crude products were purified by flash column chromatography (EtOAc (1% TEA)).

6. GC Analysis

Biotransformations were analysed using an Agilent HP6890 gas chromatograph fitted with Agilent HP-5MS Ultra Inert column (30 m x 250 μm x 0.25 μm). The temperature program was 70 °C for 2 min, then 10 °C min^{-1} until 90 °C, then 30 °C min^{-1} until 180 °C, followed by a plateau at this temperature for 6 min. H_2 flow rate: 1.2 mL min^{-1}. Inlet temperature: 250 °C. Flame ionisation detector's temperature: 300 °C.

7. Time courses of biotransformations analysed using GC

The analysis of biotransformations by IR77 and mutants gave the time courses shown in **Figure S6**.

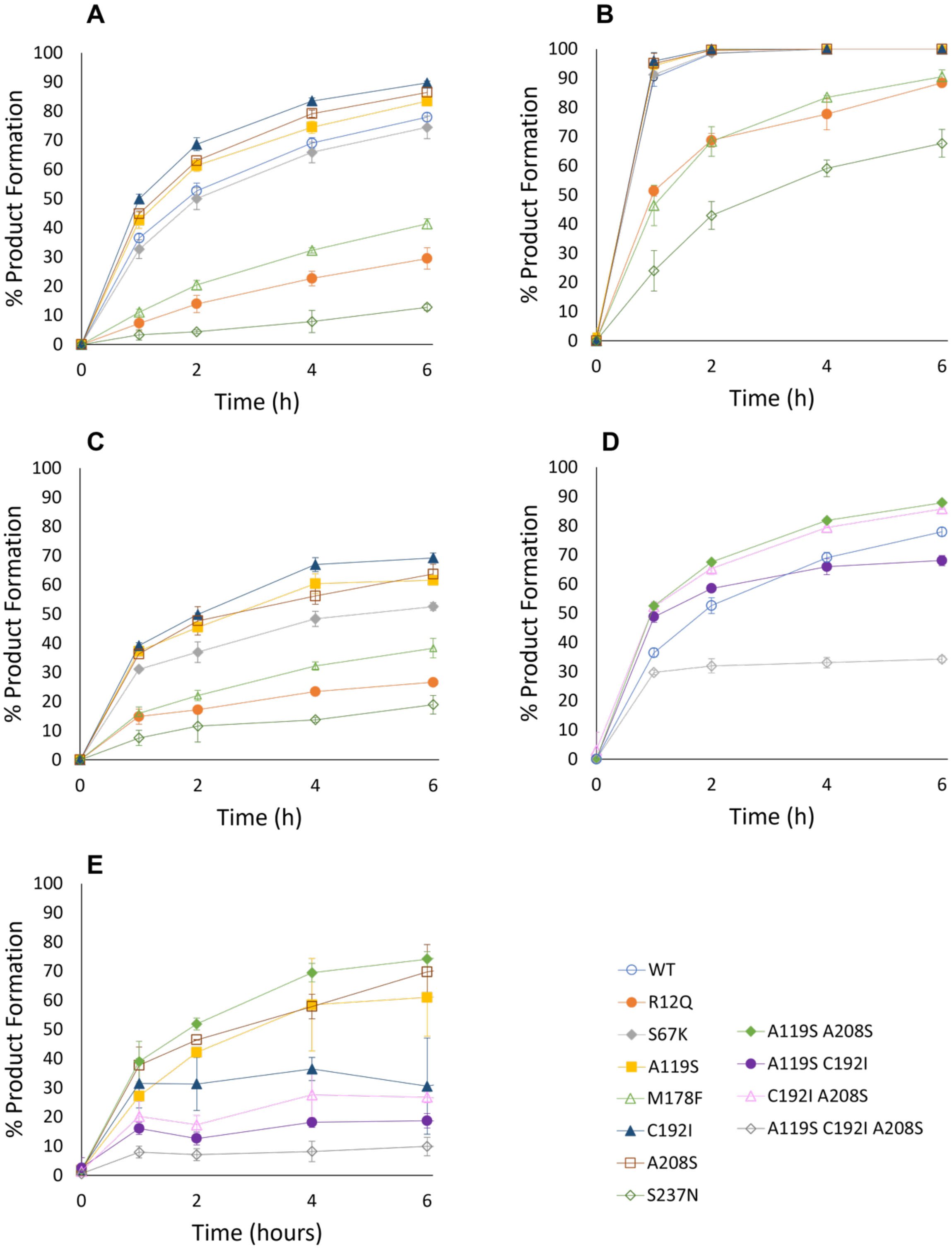

Figure S6. Plots of % product conversions over first 6 h. **A**. Reactions with cyclohexanone (10 mM) and pyrrolidine (2.5 m.e.); **B**. Reactions with cyclohexanone (10 mM) and isoindoline (2.5 m.e.); **C**. Reactions with cyclohexanone (10 mM) and 2-octahydrocyclopenta[c]pyrrole (2.5 mM); **D**. Reactions with cyclohexanone (10 mM) and pyrrolidine (2.5 m.e.) for double and triple mutants; **E**. Reactions with cyclohexanone (80 mM) and isoindoline (1.2 m.e.) with best single mutants and double and triple mutants.

8. Synthesis of Standards and Characterisation of Reaction Products

General

NMR spectra were obtained in the solvent indicated, using a JEOL ECX400 or JEOL ECS400 spectrometer (400MHz, 101 MHz and 162 MHz for ^{1}H and ^{13}C and respectively), or a Bruker 500 (500 MHz, 126 MHz and 202 MHz for ^{1}H and ^{13}C respectively). Chemical shifts are reported in parts per million and were referenced to the residual non-deuterated solvent of the deuterated solvent used ($CHCl_3$ TMH = 7.26 and TMC = 77.16 ($CDCl_3$), $(CHD_2)SO(CD_3)$ TMH = 2.50 and TMC = 39.52 $\{SO(CD_3)_2\}$, ^{1}H and ^{13}C, respectively). Spectra were typically run at a temperature of 298 K. All ^{13}C NMR spectra were obtained with ^{1}H decoupling. NMR spectra were processed using MestreNova software (Version 11.0.2-18153, released October 2016). MS spectra were measured using a Bruker Daltonics micrOTOF MS, Agilent series 1200LC with electrospray ionisation (ESI and APCI), or on a Thermo LCQ using electrospray ionisation, with <5 ppm error recorded for all HRMS samples.

Syntheses of amine standards 1a and 1f

General procedure.[2]

To a solution of cyclohexanone (**1**, 2.5 mmol, 1.0 eq.) in dry THF (10 mL) under N_2 were added the corresponding primary amine (**a** or **f**, 2.75 mmol, 1.1 eq.), $NaBH(OAc)_3$ (3.75 mmol, 1.0 eq.) and glacial acetic acid (2.5 mmol, 1.0 eq.). The reaction was stirred for 16 h at room temperature under N_2 then quenched by addition of aqueous NaOH solution (10 mL, 5.0 M). EtOAc (20 mL) was added, and the phases were separated. The aqueous phase was extracted with a further portion of EtOAc (20 mL). The organic phases were combined and extracted with 1.0 M aqueous HCl (3 × 10 mL) and the aqueous phase basified to pH 12 by the addition of aqueous NaOH solution (5.0 M). The product was extracted into EtOAc (2 × 20 mL). The combined organic phases were dried over $MgSO_4$, filtered, and concentrated *in vacuo* to afford the corresponding secondary amines **1a** and **1f**.

***N*-Benzylcyclohexanamine 1a**

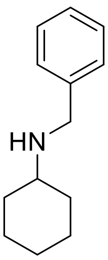

Brown oil, 109 mg, 23% yield.

^{1}H NMR (400 MHz, $CDCl_3$) δ 7.30 - 7.24 (m, 4H), 7.22 - 7.16 (m, 1H), 3.76 (s, 2H), 2.49 - 2.39 (m, 1H), 1.90 - 1.83 (m, 2H), 1.73 - 1.63 (m, 2H), 1.60 - 1.53 (m, 1H), 1.24 - 1.01 (m, 5H).; ^{13}C NMR (101 MHz, $CDCl_3$) δ 141.1, 128.5, 128.2, 126.9, 56.3, 51.2, 33.7, 26.3, 25.1.; HRMS (ESI+, m/z): calculated for $C_{13}H_{20}N$ [$(M+H)^+$]: 190.1590; found 190.1591. The data match those described in the literature.[3]

N-Cyclohexylaniline 1f

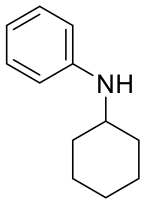

Yellow oil, 380 mg, 87% yield.

^{1}H NMR (400 MHz, $CDCl_3$) δ 7.16 (dd, J = 8.6, 7.3 Hz, 2H), 6.70 - 6.63 (m, 1H), 6.62 - 6.57 (m, 2H), 3.51 (s, 1H), 3.26 (tt, J = 10.2, 3.7 Hz, 1H), 2.11 - 2.02 (m, 2H), 1.76 (ddd, J = 9.1, 4.6, 2.4 Hz, 2H), 1.70 - 1.62 (m, 1H), 1.47 - 1.29 (m, 2H), 1.31 - 1.07 (m, 3H).; ^{13}C NMR (101 MHz, $CDCl_3$) δ 147.5, 129.4, 116.9, 113.2, 51.8, 33.6, 26.1, 25.2.; HRMS (ESI+, m/z): calculated for $C_{12}H_{18}N$ [$(M+H)^+$]: 176.1434; found 176.1434. The data match those described in the literature.[4]

Syntheses of amine standards 1b and 1e

General procedure.[2]

To a solution of cyclohexanone (**1**, 2.5 mmol, 1.0 equiv.) in MeOH (7.5 mL) were added 3 Å molecular sieves and the corresponding amine (**2**, 5.0 mmol, 2.0 eq.). The solution was stirred for 3 h at room temperature. The reaction was then cooled to 0 °C and $NaBH_4$ (5.0 mmol, 2.0 eq.) was added in portions. The reaction was then allowed to warm to room temperature and stirred for a further 0.5 h. The reaction was quenched by the addition of aqueous HCl (2.0 M) until no more evolution of gas was observed. The volatiles were removed *in vacuo* and water (7.5 mL) and EtOAc (10 mL) were added. The mixture was basified to pH 12 with aqueous NaOH solution (5.0 M) and the phases were separated. The aqueous layer was further extracted with EtOAc (2 × 10 mL) and the combined organic phases were dried over $MgSO_4$, filtered and concentrated *in vacuo*. The residue was treated with HCl solution (4.0 M in 1,4-dioxane), the precipitate was filtered, washed with diethyl ether (3 × 5 mL) and dried *in vacuo* to afford the corresponding amine hydrochloride salts.

1-Cyclohexylpyrrolidine hydrochloride, 1b

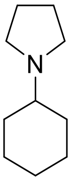

HCl

Brown solid, 50 mg, 11% yield.

^{1}H NMR (400 MHz, MeOH-d_4) δ 3.64 - 3.53 (m, 2H), 3.15 - 3.01 (m, 3H), 2.20 – 1.91 (m, 6H), 1.92 - 1.81 (m, 2H), 1.73 - 1.62 (m, 1H), 1.50 - 1.26 (m, 4H), 1.29 - 1.15 (m, 1H).; ^{13}C NMR (101 MHz, MeOH-d_4) δ 65.3, 52.6, 30.4, 26.0, 25.6, 23.9.; HRMS (ESI+, m/z): calculated for $C_{10}H_{20}N$ [(M+H)$^+$]: 154.1590; found 154.1586.

1-Cyclohexylmethylamine hydrochloride, 1e

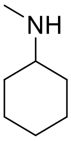

HCl

White solid, 130 mg, 35% yield.

^{1}H NMR (400 MHz, MeOH-d_4) δ 3.00 (tt, J = 7.8, 3.6 Hz, 1H), 2.67 (s, 3H), 2.11 (dq, J = 9.7, 2.9 Hz, 2H), 1.91 - 1.85 (m, 2H), 1.75 - 1.68 (m, 1H), 1.45 - 1.18 (m, 5H).; ^{13}C NMR (101 MHz, MeOH-d_4) δ 59.3, 30.5, 30.1, 26.1, 25.3.; HRMS (ESI+, m/z): calculated for $C_7H_{16}N$ [(M+H)$^+$]: 114.1277; found 114.1279.

Syntheses of Amine standards 1h and 1i

2-Cyclohexyl-isoindoline 1h

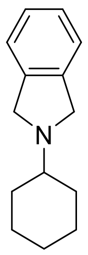

Isoindoline (223 µL, 1.97 mmol, 1.00 eq.) was added to a stirred solution of cyclohexanone (211 µL, 2.04 mmol, 1.03 eq.) in anhydrous THF (10 mL), followed by the addition of $NaBH(OAc)_3$ (660 mg, 2.83 mmol, 1.44 eq.) and glacial acetic acid (140 µL, 2.45 mmol, 1.42 eq.). The resulting slurry was stirred at room temperature overnight and it was quenched with saturated $NaHCO_3$ solution (*aq.*, 10 mL) and then extracted with EtOAc (3 × 10 mL). The combined organic fractions were extracted with aqueous HCl solution (1.0 M, 3 × 10 mL) and the resulting aqueous fractions were combined and basified with aqueous NaOH solution (5.0 M) before being extracted with EtOAc (3 × 10 mL). The organic fractions were combined, dried with $MgSO_4$, filtered and concentrated under reduced pressure to give the title compound as a brown solid (258 mg, 1.28 mmol, 65%).

^{1}H NMR (400 MHz, $CDCl_3$) δ 7.24 - 7.15 (m, 4H), 3.94 (s, 4H), 2.47 - 2.32 (m, 1H), 2.07 - 1.93 (m, 2H), 1.87 - 1.74 (m, 2H), 1.68 - 1.59 (m, 1H), 1.40 - 1.18 (m, 5H); ^{13}C NMR (100 MHz, $CDCl_3$) δ 140.1, 126.7, 122.4, 62.8, 56.9, 32.1. 26.2, 24.9. GC-MS (EI) *m/z* = 201.

2-Cyclohexyl-3,3a,4,5,6,6a-hexahydro-1H-cyclopenta[c]pyrrole hydrochloride 1i

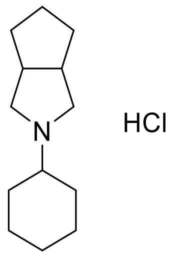

Octahydrocyclopenta[c]pyrrole hydrochloride (266 mg, 1.80 mmol, 1.00 eq.) was added to a stirred solution of cyclohexanone (211 µL, 2.04 mmol, 1.13 eq.) in anhydrous THF (10 mL) before the addition of $NaBH(OAc)_3$ (585 mg, 2.76 mmol 1.53 eq.) and glacial acetic acid (140 µL, 2.45 mmol, 1.36 eq). The resulting slurry was stirred at room temperature overnight before

being quenched with saturated $NaHCO_3$ (*aq*., 10 mL) and then extracted with EtOAc (3 × 10 mL). The combined organic fractions were extracted with aqueous HCl solution (1.0 M, 3 × 10 mL) and the resulting combined aqueous fractions were basified with aqueous NaOH solution (5.0 M) and then extracted with EtOAc (3 × 10 mL). The combined organic fractions were dried with $MgSO_4$, filtered before being acidified with HCl in 1,4-dioxane (2.0 M, 900 µL, 2.0 equiv.) and the solvent was removed by N_2 flow to give the title compound (104 mg, 0.45 mmol, 25%).

^{1}H NMR (400 MHz, $CDCl_3$) δ 3.09 – 2.96 (m, 2H), 2.66 - 2.52 (m, 2H), 2.94 - 1.77 (m, 5H), 1.77 - 1.65 (m, 2H), 1.65 - 1.44 (m, 5H), 1.44 - 1.33 (m, 2H), 1.29 - 1.04 (m, 5H); ^{13}C NMR (100 MHz, $CDCl_3$) δ 63.7, 59.4, 42.0, 32.5, 32.1, 26.3, 25.4, 25.3. GC-MS (EI) *m/z* = 193.

Characterization of Products from Scaled Biotransformations

1-Cyclohexylpyrrolidine 1b

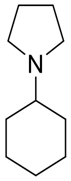

Yellow oil, 62% yield.

^{1}H NMR (400 MHz, $CDCl_3$) δ 2.62 - 2.48 (m, 4H), 2.17 - 2.03 (m, 3H), 1.81 - 1.63 (m, 6H), 1.61 - 1.51 (m, 1H), 1.29 - 1.03 (m, 5H).; ^{13}C NMR (101 MHz, $CDCl_3$) δ 64.0, 51.5, 31.9, 26.0, 25.3, 23.3. The data match those described in the literature.[5]

2-Cyclohexyl-isoindoline 1h

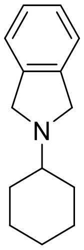

Cream feathery crystals, 93% yield.

^{1}H NMR (400 MHz, $CDCl_3$) δ 7.25 - 7.11 (m, 4H), 3.97 (s, 4H), 2.45 - 2.33 (m, 1H), 2.06 - 1.95 (m, 2H), 1.84 - 1.75 (m, 2H), 1.67 - 1.58 (m, 1H), 1.39 - 1.15 (m, 5H).; ^{13}C NMR (101 MHz, $CDCl_3$) δ 140.0, 126.7, 122.4, 62.9, 56.8, 34.0, 26.1, 24.8.

***N*-Benzylcyclohexanamine 1a**

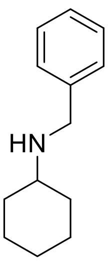

Yellow oil, 71% yield.

^{1}H NMR (400 MHz, $CDCl_3$) δ 7.36 - 7.19 (m, 5H), 3.80 (s, 2H), 2.54 - 2.42 (m, 1H), 1.97 - 1.86 (m, 2H), 1.79 - 1.68 (m, 2H), 1.66 - 1.55 (m, 1H), 1.33 - 1.05 (m, 5H).; ^{13}C NMR (101 MHz, $CDCl_3$) δ 141.1, 128.5, 128.2, 126.9, 56.3, 51.2, 33.7, 26.3, 25.1. The data match those described in the literature.[3]

9. $NaBH_3CN$ Reactions and Comparisons

Reaction Protocol for reductive amination using $NaBH_3CN$

To 3 mL of Tris-HCl buffer (100 mM, pH 9.0) in a 10 mL glass vial, were added 10 cyclohexanone from a 1 M stock in DMSO to a final concentration of 10 mM, amine (1.2 eq.) and $NaBH_3CN$ to a final concentration of 150 mM . For reactions with pyrrolidine, the 10 mL vial was shaken at 250 r.p.m at 25 ˚C for 24 h. 200 µL aliquots of this reaction were taken at the time points specified. For reactions with isoindoline, 200 µL aliquots from the 10 mL vial were dispensed into ten 1.5 mL Eppendorf tubes and these were shaken at 250 r.p.m. at 25 ˚C for 24 h. At each timepoint, one of these 200 µL aliquots was basified with 16 µL NaOH (10.0 M), after which the aqueous layer was extracted with 400 µL EtOAc, the organic phase was dried over $MgSO_4$ and the supernatant was directly subjected to GC-FID analysis.

10. NMR analysis of Imine Formation in Solution

^{13}C NMR spectra were recorded on a Bruker Avance Neo 700 MHz spectrometer, equipped with a triple-resonance N_2-cooled cryoprobe. Samples contained 160 mM cyclohexanone and 192 mM (1.2eq.) of either isoindoline or pyrrolidine in Tris-HCl buffer (100 mM, pH 9.0); 10% (v/v) D_2O was added prior to NMR data collection. Prior to ^{13}C NMR analysis, the reaction was shaken at 250 r.p.m. at 25 ˚C for 30 min. The spectral width was 237 ppm, the acquisition time was 0.8 s, and the relaxation delay was 2 s. 256 scans were collected for the sample with isoindoline, while 1024 scans were collected for pyrrolidine. Spectra were processed with 1 Hz line broadening.

11. Crystallisation, Data Collection and Structure Solution and Refinement

Protein crystallisation

Initial screening of crystallization conditions was performed using commercially available INDEX (Hampton Research), PACT premier and CSSI/II (Molecular Dimensions) screens in 96-well sitting drop trays. Optimization was carried out in a 48-well sitting-drop format to obtain crystals for X-ray diffraction studies. For co-crystallization experiments, a 0.1 M stock solution of cofactor $NADP^+$ in water was prepared.

Crystals of the IR77-$NADP^+$ complex were grown using IR77 concentrated to 50 mg mL^{-1} in 50 mM Tris-HCl buffer at pH 7.1 containing 300 mM NaCl and 10% glycerol. Crystals of

IR77-NADP$^+$ were grown at 6 °C using IR77 at 50 mg mL^{-1} in 50 mM Tris-HCl pH 7.1, 300 mM NaCl, 10% glycerol in a drop with 770 nL protein; 800 nL mother liquor containing 30 % mPEG 2000 w/v, 0.2 M KSCN; and 30 nL IR77-NADP$^+$ seed stock. The crystallisation drop contained 0.77 µL protein: 0.30 µL seed stock: 0.80 µL mother liquor comprising 30 % mPEG 2000 w/v, 0.2 M KSCN, and 2 mM NADP$^+$.

Crystals of the IR77-A208N-NADP$^+$ complex were grown at 6°C using IR77-A208N concentrated to 22 mg mL^{-1} in 50 mM Tris-HCl buffer at pH 7.1 containing 300 mM NaCl and 10% glycerol, with the addition of 6 mM NADP+ and 50 mM *N*-cyclohexylisoindoline, in a drop with 800 nL protein mixture: 800 nL mother liquor containing 20 % mPEG 3350 w/v, 3% propan-1-ol v/v, 0.1 M NaI and 0.1 M BIS-TRIS propane pH 6.5. NADP+ stock solution was made up to 100 mM in water and *N*-cyclohexylisoindoline stock solution was made up to 500 mM in DMSO.

Crystals were harvested directly into liquid nitrogen with nylon CryoLoops^{TM} (Hampton Research), using the mother liquor without any further cryoprotectant.

Data collection, structure solution and refinement

The datasets described in this report were collected at the Diamond Light Source, Didcot, Oxfordshire, U.K. on beamline I04. Data were processed and integrated using XDS[6] and scaled using SCALA[7] included in the Xia2[8] processing system. Data collection statistics are provided in **Table S6**. The crystal of IR77-NADP$^+$ was obtained in space group $P2_1$, with four molecules in the asymmetric unit; The solvent content in the crystals was 49.8%. Crystals of IR77-A208N were obtained in space group $P2_12_12_1$ with two molecules in the asu. The structure of IR77-NADP$^+$ was solved by molecular replacement using MOLREP[9] with the monomer of R-IRED-*Sr* (PDB code 5OCM[10]) as the model. The finished model was used to solve the structure of IR77-208N. The structures were built and refined using iterative cycles in Coot[11] and REFMAC,[12] employing local NCS restraints in the refinement cycles. Following building and refinement of the protein and water molecules in each structure complex, residual density was observed in the omit maps at the dimer interfaces, which could be clearly modelled as NADP$^+$. The final structures of IR77 and IR77-A208N exhibited % R_{cryst}/R_{free} values of 23.6/28.1 and 19.8/24.2 respectively. Refinement statistics for the structures are presented in **Table S6.** The Ramachandran plot for IR77-NADP$^+$ showed 99.20 % of residues to be situated in the most

favoured regions, 0.60 % in additional allowed and 0.20 % residues in outlier regions. The corresponding values for IR77-A280N were 99.60% and 0.40% with no outliers. The structures of IR77 and IR77-A208N have been deposited in the Protein Databank (PDB) with accession codes **8A3X** and **8A5Z** respectively.

Table S6: Data collection and refinement statistics for IR77. Numbers in brackets refer to data for highest resolution shells.

	**wt-IR77-NADP$^+$**	**IR77-A208N-NADP$^+$**
Beamline	I04	I03
Wavelength (Å)	0.9795	0.97628
Resolution (Å)	58.00 – 2.58 (2.69-2.58)	64.63-2.31 (2.39-2.31)
Space Group	$P2_1$	$P2_12_12_1$
Unit cell (Å)	a = 90.96, b = 57.53; c = 116.44 α = 90.00˚ β = 94.96˚ γ = 90.00˚	a = 80.57, b = 88.75; c = 93.88 α = β = γ = 90.00˚
No. of molecules in the asymmetric unit	4	2
Unique reflections	38177 (4621)	30238 (2921)
Completeness (%)	99.7 (99.6)	100.0 (100.0)
R_{merge} (%)	0.17 (1.17)	0.18 (1.04)
$R_{p.i.m.}$	0.15 (0.97)	0.05 (0.30)
Multiplicity	4.1 (4.2)	13.4 (12.6)
$<I/\sigma(I)>$	4.4 (0.9)	10.2 (1.3)
Overall *B* from Wilson plot (Å$_2$)	31	31
$CC_{1/2}$	0.99 (0.67)	1.00 (0.93)
R_{cryst}/ R_{free} (%)	23.6/28.1	19.8/24.2
r.m.s.d 1-2 bonds (Å)	0.006	0.008
r.m.s.d 1-3 angles (°)	1.50	1.57
Avge main chain B (Å^2)	56	43
Avge side chain B (Å^2)	59	47
Avge waters B (Å^2)	33	39
Avge NADP$^+$ B (Å^2)	50	39

12. Structural Alignment with *Asp*RedAm

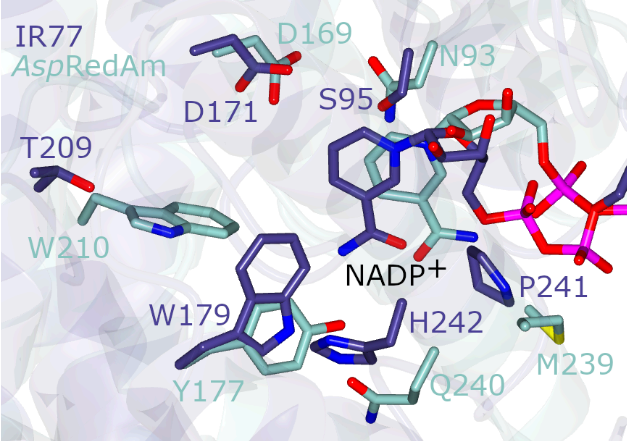

Figure S7. Superimposition of active sites of *Asp*RedAm[2] and IR77 (carbon atoms in light blue and purple respectively) and showing substitution of T209 with W210 at the rear of the active site.

13. Autodock Models

Automated docking was performed using AUTODOCK VINA 1.1.2.[13] Coordinates for the ligand were prepared using the ligand builder in COOT[11] with the relevant topology files generated in ACEDRG. The appropriate pdbqt files for the dimeric model of IR77 and the ligand were prepared in AUTODOCK Tools. The active site of IR77 was contained in a grid size of 28 Å × 28 Å × 28 Å (corresponding to x, y, z) with 1 Å spacing, centred around the catalytic centre at positions -57.78 Å × -7.35. Å × 51.87 Å (corresponding to x, y, z), which was generated using AutoGrid in the AUTODOCK Tools interface. The dockings were performed by VINA, therefore the posed dockings were below 2 Å rmsd. The results generated by VINA were visualised in AUTODOCK Tools 1.5.6 where the ligand conformations were assessed based upon lowest VINA energy.

14. Nano DSF measurements

Purified protein (1 mg mL^{-1}) in buffer (50 mM Tris-HCl, 300 mM NaCl, 10 % glycerol, pH 7.1) was loaded into high sensitivity capillaries and heated from 20 °C to 95 °C over 75 min by a Prometheus NT.48. Inflection points and first derivatives were calculated by the Prometheus NT.48 software to determine the melting temperature of each protein. For experiments with the addition of cofactor, a 100 mM stock solution of NADPH was prepared

in water and added to the proteins to a concentration of 2 mM before loading into the capillaries. Results are presented in **Table S7**.

Table S7. Melting temperatures for IR77-wt and mutants.

	T_m without addition of NADPH /°C	T_m with 2 mM NADPH Tm /°C	ΔT_m /°C
WT	44.5	50.4	5.9
A119S	41.0	45.8	4.8
C192I	36.5	41.3	4.8
A208S	41.8	48.9	7.1
A119S/A208S	40.0	47.8	7.8
A208N	41.9	50.1	8.2

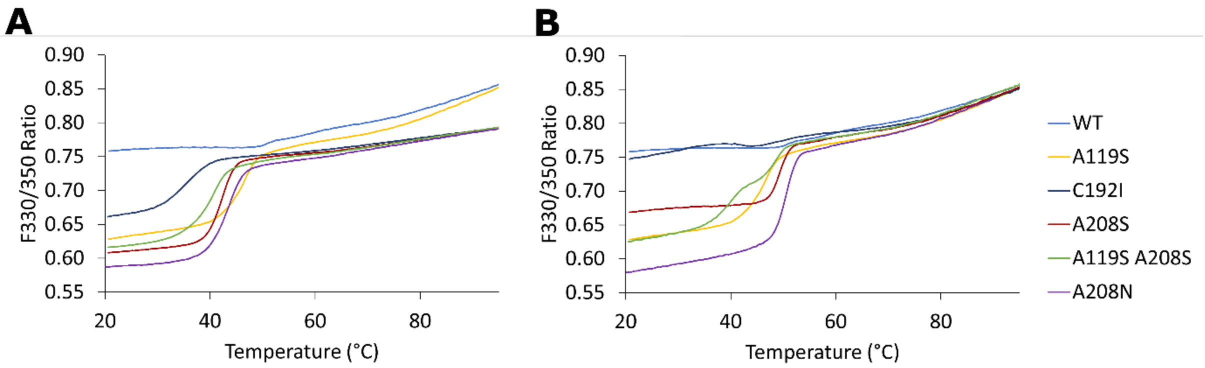

Figure S8. Melting temperature curves **A**. without added NADPH **B**. with 2mM NADPH added.

15. References

1. Schober, M.; MacDermaid, C.; Ollis, A.A.; Chang, S.; Khan, D.; Hosford, J.; Latham, J.; Ihnken, L.A.F.; Brown, M.J.B.; Fuerst, D.; Sanganee, M.J.; Roiban, G-D. Chiral Synthesis of LSD1 Inhibitor GSK2879552 Enabled by Directed Evolution of an Imine Reductase (2019) *Nat. Catal.*, **2**, 909-915.
2. Aleku, G. A.; France, S. P.; Man, H.; Mangas-Sanchez, J.; Montgomery, S. L.; Sharma, M.; Leipold, F.; Hussain, S.; Grogan, G.; Turner, N. J. A Reductive Aminase from *Aspergillus oryzae* (2017) *Nat. Chem.*, **9**, 961-969.
3. Popov, K. K.; Campbell, J. L. P.; Kysilka, O.; Hošek, J.; Davies, C. D.; Pour, M.; Kočovský, P. Reductive Amination Revisited: Reduction of Aldimines with Trichlorosilane Catalyzed by Dimethylformamide-Functional Group Tolerance, Scope, and Limitations (2022) *J. Org. Chem.* **87**, 920-943.
4. Purkait, N.; Kervefors, G.; Linde, E.; Olofsson, B. Regiospecific N-Arylation of Aliphatic Amines under Mild and Metal-Free Reaction Conditions (2018) *Angew. Chem. Int. Ed.* 2018, **57** 11427-11431.
5. Varjosaari, S.E.; Skrypai, V.; Suating, P.; Hurley, J.J.M.; De Lio, A.M.; Gilbert, T.M.; Adler, M.J. Simple Metal-Free Direct Reductive Amination Using Hydrosilatrane to Form Secondary and Tertiary Amines (2017) *Adv. Synth. Catal.* **359**, 1872-1878.
6. Kabsch, W. XDS (2010) *Acta Crystallogr. Sect. D Biol. Crystallogr.* **66**, 125-132.
7. Evans, P. Scaling and Assessment of Data Quality (2006) *Acta Crystallogr. Sect. D Biol. Crystallogr.,* **62**, 72-82.
8. Winter, G. *xia2*: An Expert System for Macromolecular Crystallography Data Reduction (2010) *J. Appl. Crystallogr.* **43**, 186-190.
9. Vagin, A.; Teplyakov, A. MOLREP: An Automated Program for Molecular Replacement (1997) *J. of Appl. Crystallogr.* **30**, 1022-1025.
10. Lenz, M.; Fademrecht, S.; Sharma, M.; Pleiss, J.; Grogan, G.; Nestl, B. M. New Imine-Reducing Enzymes from β-Hydroxyacid Dehydrogenases by Single Amino Acid Substitutions (2018) *Protein Eng. Des. Sel.* **31**, 109-120.
11. Emsley, P.; Cowtan, K., Coot: Model Building Tools for Molecular Graphics (2004) *Acta Crystallogr. Sect. D Biol. Crystallogr.* **60**, 2126-2132.

12. Murshudov, G. N.; Vagin, A. A.; Dodson, E. J. Refinement of Macromolecular Structures by the Maximum-Likelihood Method (1997) *Acta Crystallogr. Sect. D Biol. Crystallogr.* **53**, 240-255.
13. Trott, O.; Olson, A. J. AutoDock Vina: Improving the Speed and Accuracy of Docking with a New Scoring Function, Efficient Optimization and Multithreading (2009) (2009) *J. Comput. Chem.* 2009, **31**, 455-461.
